# mvlearnR and Shiny App for multiview learning

**DOI:** 10.1093/bioadv/vbae005

**Published:** 2024-01-16

**Authors:** Elise F Palzer, Sandra E Safo

**Affiliations:** Division of Biostatistics and Health Data Science, University of Minnesota, Minneapolis, Minnesota 55414, United States; Division of Biostatistics and Health Data Science, University of Minnesota, Minneapolis, Minnesota 55414, United States

## Abstract

**Summary:**

The package mvlearnR and accompanying Shiny App is intended for integrating data from multiple sources or views or modalities (e.g. genomics, proteomics, clinical, and demographic data). Most existing software packages for multiview learning are decentralized and offer limited capabilities, making it difficult for users to perform comprehensive integrative analysis. The new package wraps statistical and machine learning methods and graphical tools, providing a convenient and easy data integration workflow. For users with limited programming language, we provide a Shiny Application to facilitate data integration anywhere and on any device. The methods have potential to offer deeper insights into complex disease mechanisms.

**Availability and implementation:**

mvlearnR is available from the following GitHub repository: https://github.com/lasandrall/mvlearnR. The web application is hosted on shinyapps.io and available at: https://multi-viewlearn.shinyapps.io/MultiView_Modeling/.

## 1 Introduction

Multiple types of data or views (e.g. genomics, proteomics, metabolomics) are now frequently collected on the same individuals, leading to a new area of research called multiview learning. This approach recognizes that analyzing different types of data together can help uncover the underlying mechanisms of complex diseases, marking a shift from analyzing each type of data separately. However, analyzing these data types to obtain useful information and knowledge is challenging because the data are complex, heterogeneous, and high-dimensional, and requires a considerable level of analytical sophistication.

Several methods have been proposed to associate data from multiple sources, some of which are unsupervised, while others are a mix of supervised and unsupervised techniques (e.g. Hotelling 1936, Horst 1961, Kettenring 1971, Lock *et al.* 2013, Safo *et al.* 2021, Safo and Lu 2023). Some unsupervised methods involve learning low-dimensional representations that maximize association between different data types (e.g. Hotelling 1936, Safo *et al.* 2018), while others learn common (e.g. Wang *et al.* 2023), and both common and view-dependent low-dimensional representations (e.g. Lock *et al.* 2013). These methods are beneficial for data exploration, and when an outcome variable is available, the low-dimensional representations are then associated with the clinical outcome. The joint association and prediction methods, on the other hand, combine supervised and unsupervised techniques, linking the problems of assessing associations between the views with prediction of an outcome (Safo *et al.* 2021, Wang and Safo 2021, Moon and Lee 2022, Palzer *et al.* 2022). The objective is then to learn low-dimensional representations that can predict the outcome, making it easier to interpret the data.

Most existing software packages for multiview learning tend to be decentralized, making it difficult for users to perform comprehensive integrative analysis. The mix-omics (Rohart *et al.* 2017) package for integration offers both supervised and unsupervised methods for multiview learning. However, the methods provided in mix-omics are limited. For instance, the outcome types are either continuous or categorical, not allowing for other types of outcomes (e.g. Poisson, time-to-event). The methods do not allow for the use of prior biological information which can enhance interpretability. Importantly, users must be well versed in the R programming language, which is limiting.

We provide mvlearnR, an R software for multiview learning, which will serve as a comprehensive software for integrating data from multiple sources. The new package wraps statistical and machine learning methods and graphical tools, providing a convenient and easy data integration workflow. For users with limited programming language, we provide a Shiny Application to facilitate data integration. Currently, mvlearnR can be used to:

- Prefilter each data type via differential analysis (DA). We provide both supervised and unsupervised options for DA or for filtering out noise variables prior to performing data integration.
- Integrate data from two sources using a variant of the popular unsupervised method for associating data from two views, i.e. canonical correlation analysis (CCA).
- Predict a clinical outcome using results from CCA. We provide four outcome data distribution type (i.e. gaussian, binomial, Poisson, and time-to-event data.)
- Jointly integrate data from two or more sources and discriminate between two or more classes. We provide an additional method which allows to incorporate prior biological structure (e.g. variable–variable relationships). These methods allow to include covariates.
- Visualize results from DA or integrative analysis methods. These plots include: volcano plots, UMAP plots, variable importance plots, discriminant plots, correlation plots, relevance network plots, loadings plots, and within- and between- view biplots. These visualization tools will help unravel complex relationships in multiview data.
- Demonstrate our integration workflow via already uploaded synthetic and real molecular and clinical data pertaining to COVID-19.

The rest of this paper presents the package and Shiny App, with more details in the Supplementary Material. We organize the paper as follows. First, we discuss the implementation details of the package and web application. Then, we explain in greater detail the filtering, supervised and unsupervised integration, and visualization methods. We demonstrate mvlearnR’s use on real data and interpret the results. Finally, we discuss the limitations of the package and web application and suggest potential future directions.

## 2 Methods and implementation

In this section, we give details about the package and web application and summarize the methods implemented. In Supplementary Table S1, we provide the currently available functions in mvlearnR and their descriptions.

### 2.1 The mvlearnR web app and package

**Figure 1.**
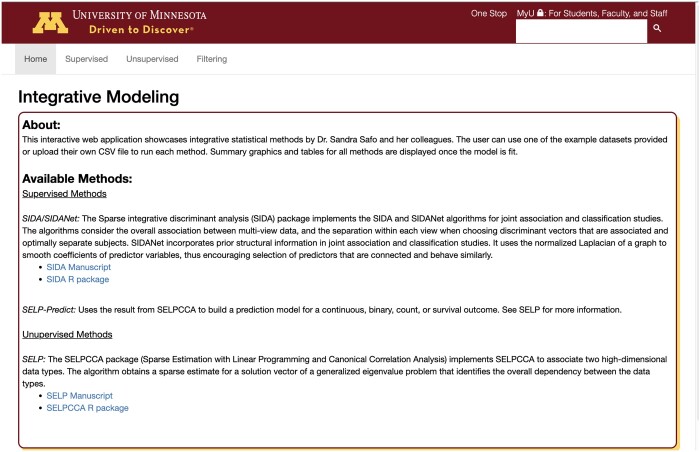
Multiview Shiny App Interface: Our Shiny App will allow non-users of R to seamlessly conduct integrative analysis. The web application has four tabs. The first tab, “Home,” provides a brief overview of the methods and related links. The second tab, “Supervised,” is where the user will implement supervised integrative analysis methods. The third tab, “Unsupervised,” is where the user will implement unsupervised integrative analysis methods. We provide options for the user to upload their own data or use example data. These tabs produce outputs of the model including classification performance, variable importance tables and plots, and several other plots to help the user understand the results. The fourth tab, “Filtering,” is where the user has the option to filter and preprocess their data to a customizable lower dimensional subset prior to data integration, using supervised and unsupervised filtering methods.

The mvlearnR web app consists of a user-friendly interface (Fig. 1), it is ideal for users with limited programming expertise in R, and it can be used anywhere and on any device. Leveraging state-of-the-art unsupervised (Safo *et al*. 2018) and supervised (Safo *et al*. 2021) integrative analysis methods, mvlearnR web server and package enable researchers to integrate molecular and clinical data, ultimately reducing the gap from raw molecular data to biological insights. The web application has four tabs. The first tab, “Home,” provides a brief overview of the methods and related links (Fig. 1). The second tab, “Supervised,” is where the user implements supervised integrative analysis methods. The third tab, “Unsupervised,” is where the user implements unsupervised integrative analysis methods. We provide options for the user to upload their own data or use example data. These tabs produce outputs of the model including classification performance, variable importance tables and plots, and several other plots to help the user understand the results. The fourth tab, “Filtering,” is where the user has the option to filter and preprocess their data to a customizable lower dimensional subset prior to data integration, using supervised and unsupervised filtering methods. The web application uses the R Shiny framework and is hosted at shinyapps.io.

In the R-package, we provide real data pertaining to COVID-19 severity and status. The data are from a study conducted by Overmyer *et al.* (2021) that collected blood samples from 102 participants with COVID-19 and 26 participants without COVID-19 and quantified for metabolomics, RNA sequencing (RNA-Seq), proteomics, and lipidomics. We provide in this package the proteomics and RNA-seq data as preprocessed in Lipman *et al.* (2022). Disease severity was measured using the World Health Organization (WHO) 0–8 disease specific scale (8 denotes death), and a score out of 45 that indicates the number of hospital free days (HFD-45) (Overmyer *et al.* 2021). These two outcome variables and other metadata (e.g. age, sex, comorbidities) are provided in the R-package. We refer the reader to Overmyer *et al.* (2021) for more details on available data. The R-package can be downloaded from GitHub at https://github.com/lasandrall/mvlearnR. We next describe the methods and functions.

### 2.2 Data import and filtering

We provide real data pertaining to COVID-19 severity and status and several simulated datasets to demonstrate the use of the package. Simulated data for two views and a binary outcome could be read into R as data(sidaData), and data(selpData). The COVID-19 data can be imported into R as data(COVIDData) (Supplementary Fig. S3). This is a list with three entries: Proteomic, RNASeq, and Clinical data. Integrative analysis methods sometimes perform poorly on large datasets so we provide supervised and unsupervised methods to filter data to help users focus on variables that are more likely to yield meaningful findings after integration. In the R-package, the function filter.supervised() (Supplementary Fig. S5) can be used to filter each view when an outcome is available via the four methods: linear, logistic, t-test, and Kruskal-Wallis (KW) test. Supervised filtering allows the user to filter variables based on their association with an outcome. *P*-values can be adjusted for multiple hypothesis testing. The function filter.unsupervised() can be used to filter each view using unsupervised methods such as variance and interquartile range (IQR) filtering. We provide an option to log2 transform variables, scale variables to have variance one, center variables to have mean zero, or normalize variables to have mean zero and variance one. Quality control checks (e.g. batch correction) and other form of normalizations specific to a particular omics data should be done outside mvlearnR.

The web application provides the filtering, scaling, centering, and normalization options. Regarding the data to be uploaded for filtering, the user can upload (i) Train and Test Sets, for when uploaded data have already been split into training and testing sets. Filtering will be conducted only on the training data. After filtering is complete, a new test set will be constructed to contain the same variables as the filtered training data; (ii) Full dataset, for when uploaded data have not been split into training and testing sets. If the user would like the app to create separate training and testing sets, we provide an option for this via the “Pct in Training set” tab.

### 2.3 Unsupervised methods for associating data from two sources

We provide the sparse canonical correlation analysis (CCA) method, SELPCCA, proposed in Safo *et al.* (2018), and described in details in the Supplementary Material for unsupervised data integration. CCA (Hotelling 1936) is a multivariate linear dimension reduction method for maximizing association between data from two sources. CCA finds weighted combinations of variables in each data that maximize the overall dependency structure between pairs of data. In the context of data integration strategies described in Picard *et al.* (2021) (e.g. early, intermediate, and late data integration), CCA falls under the intermediate strategy. The classical CCA finds linear combinations of all available variables, and since these weights are typically nonzero, it is difficult to interpret the findings. SELPCCA (Safo *et al.* 2018) is a variant of CCA that shrinks some of the weights of the low-dimensional representations to zero, thus allowing to identify relevant variables contributing to the overall dependency structure in the data. SELPCCA is thus a feature extraction and feature selection method, falling under the intermediate strategy umbrella for data integration. The function cvselpscca() (Supplementary Fig. S8) can be used to obtain low-dimensional linear representations that maximize associations between pairs of data, and to identify key variables contributing to the maximum correlation between the pairs of data. The main inputs to cvselpscca() are the train data and the number of canonical vectors, “ncancorr,” to be estimated, which defaults to 1 if not specified.

The output of cvselpscca() include: “hataplha” and “hatbeta,” representing the loadings or canonical vectors for the two views, respectively; “optTau,” the optimal tuning parameter for each data type, and “maxcorr,” estimated canonical correlation coefficient, which shows the strength of the association between the data types. The canonical vectors could be visualized, for more insight (see Section on Visualizations and Supplementary Material). Since these loadings are standardized to have unit norm, a variable with larger weight contributes more to the association between the views. Please refer to the Section Use Case for a demonstration of the cvselpscca() function.

On the web application, the SELPCCA method is located on the “Unsupervised” tab. The user can upload their data and then use the default hyper parameters to obtain the canonical vectors. After hitting the “Run Model” tab, a notification button notifies the user that the model is running. After completion, the top 20 selected variables from each view is printed out.

### 2.4 Prediction with learned low-dimensional representations from unsupervised methods

Since SELPCCA is an unsupervised method, it can only be used to identify variables contributing to the maximal association between two views. SELPCCA is ideal as an exploratory method to explore variables that contribute to the overall dependency structure between the views. If an outcome is available, one can associate the learned low-dimensional representation(s) with the outcome. We provide the function selpcca.pred() (Supplementary Fig. S15) for this purpose where the results from the SELPCCA model are used to build a generalized linear model (GLM) or Cox prediction model. The required option “family” is a string specifying the type of prediction model to build. Options are gaussian, binomial, Poisson, and survival. When family = “survival,” a Cox proportional model will be fitted. Otherwise a GLM will be used. The function predict() can be used to predict out of sample data using the learned low-dimensional representations (Supplementary Fig. S16). The performance of these new predictions can be assessed on a training data using the function PerformanceMetrics() (Supplementary Fig. S16). Currently two family options are provided: “binomial” and “gaussian.”

On the web application, the SELP-Predict method is located on the “Supervised” tab. The results from the SELPCCA model are used to build a generalized linear model (GLM) or Cox prediction model. To implement this function, the user uploads their data, sets the distribution family, and can use the default hyper parameters to obtain the canonical variates. The required option “family” is a string specifying the type of prediction model to build. Options are gaussian, binomial, poisson, and survival. When family = “survival,” a Cox proportional model will be fitted. Otherwise a GLM will be used. After model implementation, the user can view the model estimates, obtain some prediction estimates, and visualize the top 20 selected variables for each view.

### 2.5 Supervised methods for associating data from two or more sources

Sparse integrative discriminant analysis [SIDA] (Safo *et al.* 2021) is an integrative analysis method for jointly modeling associations between two or more views and creating separation of classes within each view. The algorithm considers the overall association between multiview data, and the separation within each view when choosing discriminant vectors that are associated and optimally separate subjects. Thus, SIDA combines the advantages of linear discriminant analysis (Hotelling 1936), a supervised learning method for maximizing separation between classes in one view, and CCA, an unsupervised learning method for maximizing correlation between two data types, and falls under the intermediate strategy for data integration (Picard *et al.* 2021). SIDA allows the user to select key variables that contribute to the maximum association of the views and separation of the classes. The function cvSIDA() performs n-fold cross-validation to select optimal tuning parameters for SIDA based on training data, and predicts training or testing class membership. The function cvSIDANet() incorporates prior structural information (e.g. gene-gene connectivity) in SIDA via the use of the normalized Laplacian of a graph, thus encouraging selection of predictors that are connected and behave similarly. This enhances interpretability. Covariates, if available, can be included, via the option WithCovariates==TRUE.

## 3 Visualizations

Results from the supervised filtering approach could be visualized via volcano plots using the function volcanoPlot(). The filtered or original data could be visualized via uniform manifold approximation projection [UMAP] (McInnes *et al.* 2018) with the function umapPlot(). We provide the function VarImportancePlot() to visualize the weights (in absolute value) of the low-dimensional loadings from cvselpscca(), cvSIDA(), and cvSIDANet(). Since the low-dimensional loadings are standardized to have unit norm, a variable with larger weight contributes more to the association between the views (for the unsupervised integrative analysis methods) or to the association between the views and the discrimination of classes within each view (for the supervised integrative analysis methods). We provide the function DiscriminantPlots() and CorrelationPlots() to visualize the class separation within each view, and correlations between pairs of views, respectively. We provide the function networkPlot() for relevance network that shows variable–variable connections between pairs of views. We provide the function LoadingsPlot() to visualize the loadings for each view and to demonstrate the relationships between pairs of variables within each view. We provide the function WithinViewBiplot() to show the scores and loadings together for a specific view. The function BetweenViewBiplot() shows the scores and loadings from pairs of views together.

## 4 Use case

### 4.1 Demonstration of SELPCCA

In this Subsection, we demonstrate the use of SELPCCA on multiomics data pertaining to COVID-19. Our goal is to associate Proteomics and RNASeq data to identify proteins and genes driving the overall dependency structure between the two molecular data. We then associate the canonical variates with COVID-19 status in a logistic regression model to investigate whether the canonical variates are able to discriminate between individuals with and without COVID-19.

We load the data in as data(COVIData) (Supplementary Fig. S3). The number of cases (COVID-19) and non-cases (non-COIVD-19) is 98 and 22, respectively. There are 264 proteins and 5800 genes. In this analysis, View 1 corresponds to the proteomis data, and View 2, the RNASeq data. We subset the data into 90% training, and 10% testing, keeping the proportion of cases and non-cases similar to the proportion in the whole data (Supplementary Fig. S4). We filter data using the function filter.supervised() (Supplementary Fig. S5) with the options: “method” = “logistic”; “padjust” = TRUE; “adjmethod" = BH; “standardize” = TRUE. Our outcome is disease status (“DiseaseStatus.Indicator”). After univariate filtering by removing proteins and genes that are not statistically significant (adjusted *P*-value > .05), 87 proteins and 2573 genes remain. We use the function volcano() to obtain volcano plots for proteins and genes (Supplementary Fig. S6). We use the function umapPlot() to obtain UMAP plots of the filtered data to visualize how well the samples are separated (Supplementary Fig. S7).

To fit SELPCCA, we invoke the function cvselpscca() and set the number of canonical variates to 2 (Supplementary Fig. S8). From running SELPCCA, we observed that 78 proteins and 32 genes have nonzero coefficients on the first CCA vector, which suggests that these proteins and genes maximize correlation between the proteomics and RNASeq data (estimated correlation is 0.636). Further, 54 proteins and 9 genes have nonzero coefficients on the second CCA vector, with estimated correlation 0.599. The top 20 proteins (shown as Uniprot IDs, UID) and genes with highest absolute loadings for the first CCA vector are shown in Supplementary Fig. S9. These figures are obtained with the function VarImportancePlot(). Some of the highly ranked proteins for the first CCA vector include: Immunoglobulin lambda variable 3–1 (UID P01715), HLA class I histocompatibility antigen, B alpha chain (UID P30491), Alpha-2-HS-glycoprotein (UID P02765). Some of the highly ranked genes on the first CCA vector include: ubiquitin conjugating enzyme E2 C (UBE2C), cell division cycle 6 (CDC6), cyclin A2 (CCNA2), and DEP domain containing 1B (DEPDC1B). We note that some of the highly ranked proteins (e.g. HLA class I histocompatibility antigen, B alpha chain [UID P30491] and Surfactant, pulmonary-associated protein B, isoform CRA_a [UID D6W5L6]) and genes (e.g. CDC6 and CCNA2) each had high log-odds ratios for discriminating between COVID-19 cases and non-cases.

We observe that the first canonical variate for each view is able to separate those with and without COVID-19 in the train (Supplementary Fig. S10) and test (Supplementary Fig. S11) sets. We use the function WithinViewBiplot() to visualize the sample discrimination, the canonical loadings for each view, and assess how the top variables in each view are related to each other (Supplementary Fig. S12). The protein Immunoglobulin lambda 4–69 (UID A0A075B6H9) appears to be highly correlated with the protein Ferritin light chain (UID P02792). The gene UBE2C is loaded on the first CCA vector, and the genes UPK3A and GPR35 are loaded on the second CCA vector. We use between-view biplots to visualize biplots for both views (Supplementary Fig. S13). This plot allows us to assess how genes and proteins are related. Solid black vectors represent loading plots for the first view (proteins). Dashed red vectors represent loadings plot for the second view (genes). We generate this plot with the function BetweenViewBiplot(). The protein Immunoglobulin lambda variable 3–1 (UID P01715) appears to be positively correlated with the gene UBE2C.

**Figure 2.**
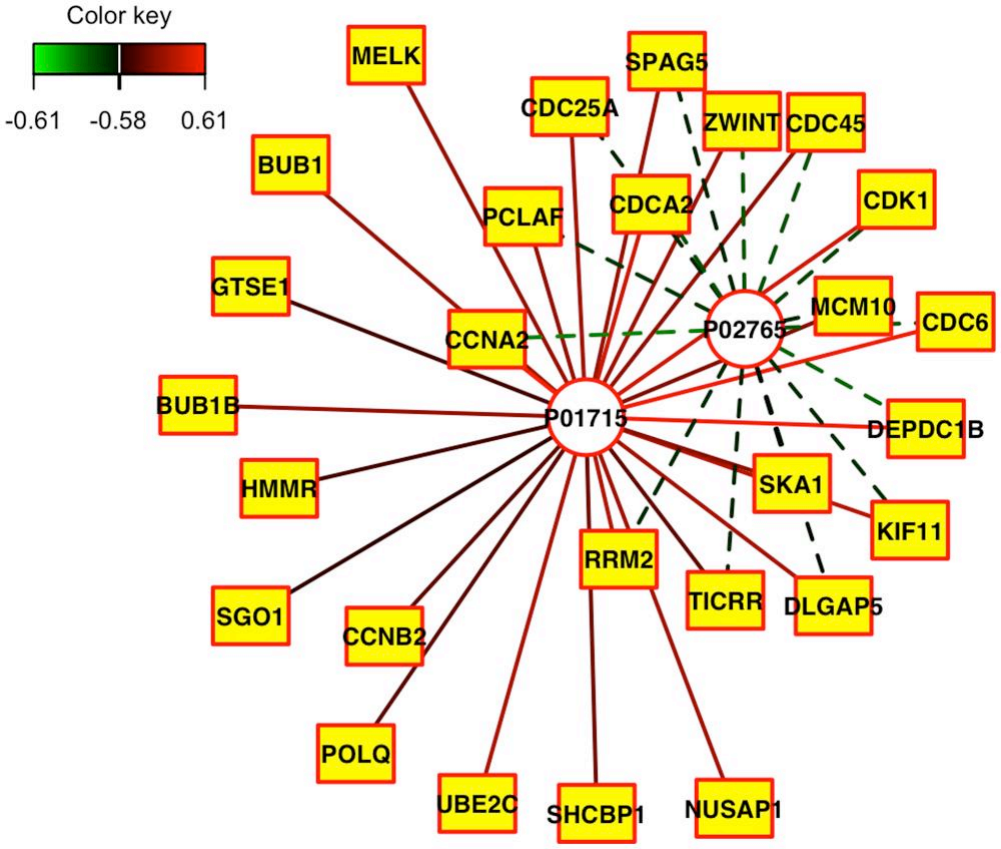
Relevance network plot for SELPCCA. The nodes of the graph represent variables for the pairs of views, and edges represent the correlations between pairs of variables. Dashed and solid lines indicate negative and positive correlations, respectively. Circle nodes are View 1 variables (proteins), and rectangular nodes are View 2 variables (genes). We show edges with correlations at least 0.58. The plot suggest that the protein P01715 is highly positively correlated with many genes, and the protein P02765 is highly negatively correlated with many genes.

For more insight into the association between the genes and proteins, we invoke the relevance network plot function networkPlot() (Fig. 2 and Supplementary Fig. S14). The nodes of the graph represent variables for the pairs of views, and edges represent the correlations between pairs of variables. Dashed and solid lines indicate negative and positive correlations, respectively. Circle nodes are View 1 variables (proteins), and rectangular nodes are View 2 variables (genes). We show edges with correlations at least 0.58. The plot suggests that the protein Immunoglobulin lambda variable 3–1 (UID P01715) is highly positively correlated with many genes (including CDC6, CCNA2, UBE2C), and the protein Alpha-2-HS-glycoprotein (UID P02765) is highly negatively correlated with many genes (including CCNA2 and CDC6).

In terms of prediction, we fitted a logistic regression model on the training data with the predictors as the first two canonical variates. We used the function selpscca.pred() for this purpose (Supplementary Fig. S15). Our results suggest that the first canonical variates for proteins and genes are significantly associated with COVID-19 status (*P*-value < .05). We predicted the test data from the learned model with the predict() function, and obtained train (Supplementary Fig. S16) and test (Supplementary Fig. S17) prediction estimates (e.g. accuracy, sensitivity, F1, etc.) with the PerformanceMetrics() function. In Supplementary Figs S15 and S16, we observe that both train and test accuracy and F1 score are high, suggesting that the first two canonical variates potentially discriminate those with and without COVID-19.

### 4.2 Demonstration of SIDA

Unlike SELPCCA which is an unsupervised method for integrating data from multiple sources, SIDA is a supervised data integration method. We demonstrate the use of SIDA on multiomics data pertaining to COVID-19. Our goal is to associate the proteomics and RNASeq data and discriminate between COVID-status in a joint model. We further identify proteins and genes that maximize both association and discrimination. We apply the function cvSIDA() (Supplementary Fig. S18) to obtain estimated SIDA discriminant vectors, correlation coefficients, and variables potentially contributing to the association of the views and the discrimination between samples within each view.

From implementing SIDA, we observed that 26 proteins and 23 genes have nonzero coefficients, which suggests that these proteins and genes maximize both correlation between the proteomics and RNASeq data (estimated correlation from train data is 0.42) as well as separation between those with and without COVID-19. The top 20 proteins (shown as Uniprot IDs, UID) and genes with highest absolute loadings are shown in Supplementary Fig. S19. Some of the highly ranked proteins include: (UID P04196), (UID P14543), (UID E9PEK4). Some of the highly ranked genes include: (GOLGA8Q), (ADGB), (TNFRSF6B), and (SLC25A41).

**Figure 3.**
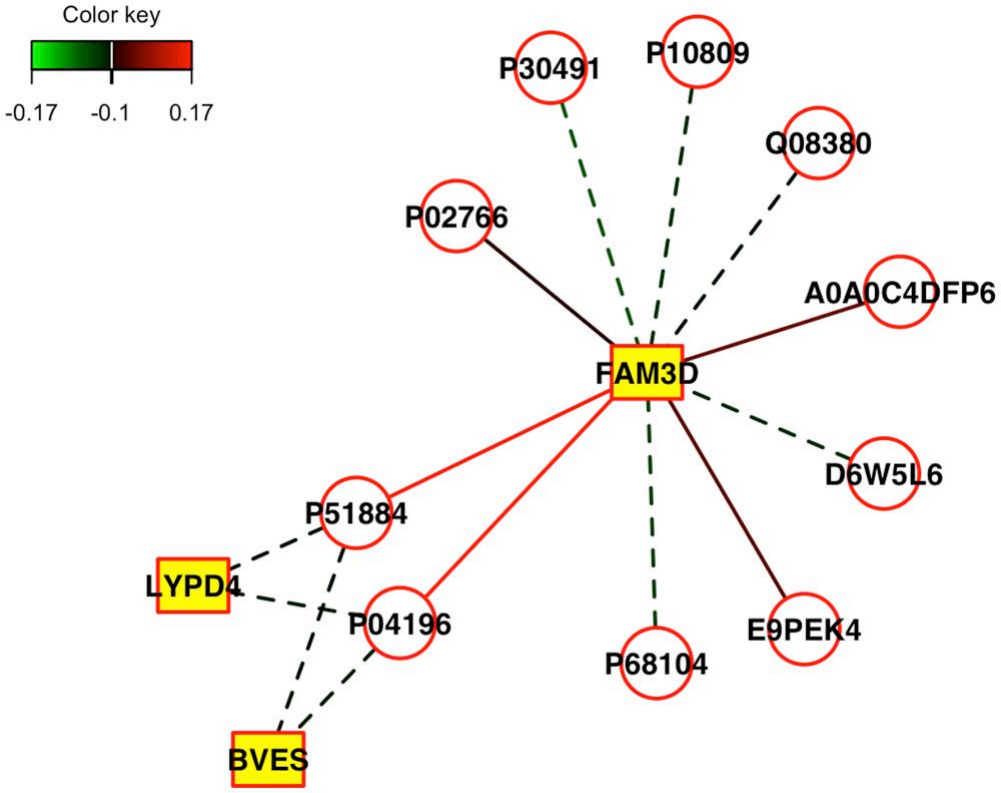
Relevance network plot for SIDA. The nodes of the graph represent variables for the pairs of views, and edges represent the correlations between pairs of variables. Dashed and solid lines indicate negative and positive correlations, respectively. Circle nodes are View 1 variables (proteins), and rectangular nodes are View 2 variables (genes). We show edges with correlations at least 0.1. The plot suggests that the gene FAM3D is negatively correlated with many proteins (e.g. PO2766, P30491, Q08380), and positively correlated with proteins such as A0ADC4DFP6, E9PEK4, P04196, and D6W5L6.

We use the function DiscriminantPlots() (Supplementary Fig. S20) to visualize the separation of COVID-19 cases and non-cases. From Supplementary Figs S20 and S21, the classes are well-separated in both the training (Supplementary Fig. S20) and testing sets (Supplementary Fig. S21). We use the function CorrelationPlots() to visualize the strength of the association between the proteins and genes and separation as well. From Supplementary Fig. S22, we notice that the views are moderately correlated, and the classes are well-separated. For more insight into the association between the genes and proteins, we invoke the relevance network plot function networkPlot() (Fig. 3 and Supplementary Fig. S23). The plot suggests that the gene FAM3D is negatively correlated with many proteins (e.g. PO2766, P30491, Q08380), and positively correlated with proteins that include A0ADC4DFP6, E9PEK4, P04196, and D6W5L6.

In terms of prediction, we obtain the train and test error upon running cvSIDA (Supplementary Fig. S18). We used the function PerformanceMetrics() to obtain other performance metrics. In Supplementary Figs S24 and S25, we observe that both train and test performance metrics are high, suggesting that SIDA discriminant scores are able to discriminate those with and without COVID-19. The estimated train correlation is 0.41. Further, the performance metrics, especially test performance metrics, are better for SIDA than SELPCCA, which suggests that in this application, joint modeling of association and separation is better.

## 5 Discussion and future work

We have introduced an R package, mvlearnR for integrating data form multiple sources. The package wraps statistical and machine learning methods and graphical tools, providing a convenient and easy data integration workflow. For users with limited programming language, we provide a Shiny Application to facilitate data integration. Our multiview dashboard will enable easy, user-friendly comprehensive integrative analysis of molecular and clinical data from anywhere and on any device, without needing to know the R language. We offer a friendly web user interface using the R Shiny framework where users can integrate multiple datasets, visualize and download results in easy to use format. Currently, linear multivariate methods for integrative analysis and biomarker identification are provided in mvlearnR, and the methods can only be used for integrating cross-sectional data. However, we have developed integrative analysis methods for disease subtyping (Zhang *et al.* 2022) and for biomarker identification where we model nonlinear relationships between data from multiple sources and a clinical outcome (Wang and Safo 2021, Safo and Lu 2023, Wang *et al.* 2023). We have also proposed a pipeline for integrating cross-sectional and longitudinal data from multiple sources (Jain and Safo 2023). These methods, and other methods we develop in the future, will eventually be added to mvlearnR and the accompanying web application. Thus, we envision mvlearnR and our web application to be a one-stop place for comprehensive data integration, for both users of R (or Python) and non-users of these software.

## Supplementary Material

vbae005_Supplementary_Data

## Data Availability

The COVID-19 molecular and clinical data used to demonstrate our integration workflow were obtained from https://doi.org/10.1016/j.cels.2020.10.003. Pre-processed data follow approach described in https://doi.org/10.1371/journal.pone.0267047.
