# Supplementary material for: mvlearnR and Shiny App for multiview learning
Source: Bioinform Adv. 2024 Jan 16;4(1):vbae005. doi: 10.1093/bioadv/vbae005 (PMC10833139; doi:10.1093/bioadv/vbae005)
Supplement: vbae005_Supplementary_Data [file vbae005_supplementary_data.pdf]

Supporting Information for `mvlearnR` for multiview learning

Elise Palzer and Sandra E Safo

January 10, 2024

The package `mvlearnR` and accompanying Shiny App is intended for integrating data from multiple sources (e.g. genomics, proteomics, metabolomics). Most existing software packages for multiview learning are decentralized, making it difficult for users to perform comprehensive integrative analysis. The new package wraps statistical and machine learning methods and graphical tools, providing a convenient and easy data integration workflow. For users with limited programming language, we provide a Shiny Application to facilitate data integration. The methods have potential to offer deeper insights into complex disease mechanisms. Currently, `mvlearnR` can be used for the following:

- Prefiltering of each omics data via differential analysis (DA). We provide both supervised and unsupervised options for DA or for filtering out noise variables prior to performing data integration.
- Integrating data from two sources using a variant of the popular unsupervised method for associating data from two views, i.e. canonical correlation analysis (CCA).
- Predicting a clinical outcome using results from CCA. We provide four outcome data distribution type (i.e. gaussian, binomial, Poisson, and time-to-event data.)
- Supervised integrative analysis (one-step) for jointly associating data from two or more sources and classifying an outcome. This method allows to include covariates.
- Supervised integrative analysis (one-step) for jointly associating structured data from two or more sources and classifying an outcome. This method allows to include covariates.
- Visualizatiing results from the DA or our integrative analysis methods. These plots include: volcano plots, UMAP plots, variable importance plots, discriminant plots, correlation plots, relevance network plots, loadings plots, and within- and between-view biplots. These visualization tools will help unravel complex relationships in the data.
- Demonstrating our integration workflow via already uploaded synthetic and real molecular and clinical data pertaining to COVID-19.

Currently, linear multivariate methods for integrative analysis and biomarker identification are provided in `mvlearnR`. However, we have developed integrative analysis methods for disease subtyping (Zhang et al., 2022) and nonlinear integrative analysis methods for biomarker

identification (Jain and Safo, 2023, Safo and Lu, 2023, Wang et al., 2023, Wang and Safo, 2021) that will eventually be added to mvlearnR and the accompanying web application. Other methods we develop in the future will be added. Thus, we envision mvlearnR and our web application to be a one-stop place for comprehensive data integration, for both users of R (or Python) and non-users of these software.

1 The Methods

The methods SELPCCA and SIDA in mvlearnR have been described in Safo et al. (2018) and Safo et al. (2021), respectively. For completeness sake, we describe these methods below. Since these methods are extensions of canonical correlation analysis (CCA)(Hotelling, 1936) and linear discriminant analysis (LDA) (Hotelling, 1936), we briefly describe these two methods.

1.1 Linear Discriminant Analysis

Suppose we have one set of data $\mathbf{X}$ with n samples on the rows and p variables on the columns. Assume the n samples are partitioned into K classes, with n_k being the number of samples in class $k, k = 1, \ldots, K$. Assume each sample belongs to one of the K classes and let y_i be the class membership for the ith subject. Let $\mathbf{X}_k = (\mathbf{x}_{1k}, \ldots, \mathbf{x}_{n_k,k})^{\mathrm{T}} \in \Re^{n_k \times p}$, $\mathbf{x}_k \in \Re^p$ be the data matrix for class k. Given these available data, we wish to predict the class membership y_j of a new subject j using their high-dimensional information $\mathbf{z}_j \in \Re^p$. Fishers linear discriminant analysis (LDA) may be used to predict the class membership. For a K class prediction problem, LDA finds $K-1$ low-dimensional vectors (also called discriminant vectors), which are linear combinations of all available variables, such that projected data onto these discriminant vectors have maximal separation between the classes and minimal separation within the classes. The within-class and between-class separation are defined respectively as: $\mathbf{S}_w = \sum_{k=1}^{K}\sum_{i=1}^{n}(\mathbf{x}_{ik} - \hat{\boldsymbol{\mu}}_k)(\mathbf{x}_{ik} - \hat{\boldsymbol{\mu}}_k)^{\mathrm{T}}$; $\mathbf{S}_b = \sum_{k=1}^{K} n_k(\hat{\boldsymbol{\mu}}_k - \hat{\boldsymbol{\mu}})(\hat{\boldsymbol{\mu}}_k - \hat{\boldsymbol{\mu}})^{\mathrm{T}}$. Here, $\hat{\boldsymbol{\mu}}_k = (1/n_k)\sum_{i=1}^{n_k}\mathbf{x}_{ik}$ is the mean vector for class k, $\hat{\boldsymbol{\mu}}$ is the combined class mean vector and is defined as $\hat{\boldsymbol{\mu}} = (1/n)\sum_{k=1}^{K} n_k\hat{\boldsymbol{\mu}}_k$. The solution to the LDA problem is given are the eigenvalue-eigenvector pairs $(\widehat{\lambda}_k, \widehat{\boldsymbol{\beta}}_k)$, $\widehat{\lambda}_1 > \cdots > \widehat{\lambda}_k$ of $\mathbf{S}_w^{-1}\mathbf{S}_b$ for $\mathbf{S}_w \succ 0$. Note that LDA is only applicable to one set of data.

1.2 Canonical Correlation Analysis

Canonical correlation analysis on the other hand is applicable when there are two views. Now, suppose we have two sets of data. $\mathbf{X}^1 = (\mathbf{x}_1^1, \cdots, \mathbf{x}_n^1)^{\mathrm{T}} \in \Re^{n\times p}$ and $\mathbf{X}^2 = (\mathbf{x}_1^2, \cdots, \mathbf{x}_n^2)^{\mathrm{T}} \in \Re^{n\times q}$, $p, q > n$, all measured on the same set of n subjects. The goal of CCA (Hotelling, 1936) is to find linear combinations of the variables in $\mathbf{X}^1$, say $\mathbf{u} = \mathbf{X}^1\boldsymbol{\alpha}$ and in $\mathbf{X}^2$, say $\mathbf{v} = \mathbf{X}^2\boldsymbol{\beta}$, such that the correlation between these linear combinations is maximized, i.e $\rho = \max cor(\mathbf{u}, \mathbf{v}) = \max cor(\mathbf{X}^1\boldsymbol{\alpha}, \mathbf{X}^2\boldsymbol{\beta})$. But

$$\max_{\boldsymbol{\alpha},\boldsymbol{\beta}} cor(\mathbf{X}^1\boldsymbol{\alpha}, \mathbf{X}^2\boldsymbol{\beta}) = \frac{\boldsymbol{\alpha}^{\mathrm{T}}\boldsymbol{\Sigma}_{12}\boldsymbol{\beta}}{\sqrt{\boldsymbol{\alpha}^{\mathrm{T}}\boldsymbol{\Sigma}_{11}\boldsymbol{\alpha}}\sqrt{\boldsymbol{\beta}^{\mathrm{T}}\boldsymbol{\Sigma}_{22}\boldsymbol{\beta}}},$$

where $\mathbf{\Sigma}_{12}$ is the $p \times q$ population covariance between $\mathbf{X}^1$ and $\mathbf{X}^2$, and $\mathbf{\Sigma}_{11}, and \mathbf{\Sigma}_{22}$ are the population covariances of $\mathbf{X}^1$ and $\mathbf{X}^2$, respectively. The population cross-covariances $\mathbf{\Sigma}_{12}$, $\mathbf{\Sigma}_{11}$, and $\mathbf{\Sigma}_{22}$ are estimated by their sample versions $\mathbf{S}_{12}$, $\mathbf{S}_{11}$, and $\mathbf{S}_{22}$, respectively. For low-dimensional settings, these sample versions are consistent estimators of the population covariances, for fixed p and q, and large n. However, for high-dimensional settings, these sample versions are regularized. The vectors $\mathbf{u}$ and $\mathbf{v}$ are called the first canonical variates for $\mathbf{X}^1$ and $\mathbf{X}^2$, respectively. We note that the correlation coefficient is invariant to scaling, thus one can choose the denominator be one and then consider the equivalent problem:

$$\max_{\boldsymbol{\alpha},\boldsymbol{\beta}} cor(\mathbf{X}^1\boldsymbol{\alpha}, \mathbf{X}^2\boldsymbol{\beta}) = \boldsymbol{\alpha}^{\mathrm{T}}\mathbf{\Sigma}_{12}\boldsymbol{\beta} \ \text{ s.t } \ \boldsymbol{\alpha}^{\mathrm{T}}\mathbf{\Sigma}_{11}\boldsymbol{\alpha} = 1, \quad \boldsymbol{\beta}^{\mathrm{T}}\mathbf{\Sigma}_{22}\boldsymbol{\beta} = 1.$$

Using Langragian multipliers, it can be shown that the CCA solution to this optimizaiton problem is given by $\widehat{\boldsymbol{\alpha}} = \mathbf{S}_1^{-1/2}\mathbf{e}_1, \widehat{\boldsymbol{\beta}} = \mathbf{S}_2^{-1/2}\mathbf{f}_1$, where $\mathbf{e}_1$ and $\mathbf{f}_1$ are the first left and right singular vectors of $\mathbf{S}_1^{-1/2}\mathbf{S}_{12}\mathbf{S}_2^{-1/2}$ respectively. We note that maximizing the correlation is equivalent to maximizing the square of the correlation. To obtain subsequent canonical variates, one can impose the following orthogonality constraints in the optimization problem: $\boldsymbol{\alpha}_i^{\mathrm{T}}\mathbf{\Sigma}_{11}\boldsymbol{\alpha}_j = \boldsymbol{\beta}_i^{\mathrm{T}}\mathbf{\Sigma}_{22}\boldsymbol{\beta}_j = \boldsymbol{\alpha}_i^{\mathrm{T}}\mathbf{\Sigma}_{12}\boldsymbol{\beta}_j = 0$, for $i \neq j$, $i, j = \min(p, q, n)$.

1.3 Sparse CCA via SELP (SELPCCA)

The classical CCA method finds linear combinations of all available variables, and since these weights are typically nonzero, it is difficult to interpret the findings. SELPCCA (Safo et al., 2018) is a variant of CCA that shrinks some of the weights of the low-dimensional representations $\boldsymbol{\alpha}$ and $\boldsymbol{\beta}$ to zero, thus allowing to identify relevant variables contributing to the overall dependency structure in the data. In particular, the authors in Safo et al. (2018) proposed to solve the following optimization problem:

$$\begin{aligned} &\min_{\boldsymbol{\alpha}} \|\boldsymbol{\alpha}\|_1 \quad s.t \quad \|\mathbf{S}_{12}\tilde{\boldsymbol{\beta}}_1 - \tilde{\rho}_1\tilde{\mathbf{S}}_{11}\boldsymbol{\alpha}\|_\infty \leq \tau_1 \\ &\min_{\boldsymbol{\beta}} \|\boldsymbol{\beta}\|_1 \quad s.t \quad \|\mathbf{S}_{21}\tilde{\boldsymbol{\alpha}}_1 - \tilde{\rho}_1\tilde{\mathbf{S}}_{22}\boldsymbol{\beta}\|_\infty \leq \tau_2. \end{aligned} \tag{1}$$

Here, $(\tilde{\boldsymbol{\alpha}}_1, \tilde{\boldsymbol{\beta}}_1)$ are the first nonsparse solution to the CCA optimization problem, and $\tilde{(\rho)}_1$ is the corresponding eigenvalue. Of note, $\tilde{\mathbf{S}}_{11}$ and $\tilde{\mathbf{S}}_{22}$ are regularized versions of $\mathbf{S}_{11}$ and $\tilde{\mathbf{S}}_{22}$, respectively. The authors considered two forms of regularizations: $\tilde{\mathbf{S}}_{11} = \mathbf{I}_p$ and $\tilde{\mathbf{S}}_{22} = \mathbf{I}_q$, for standardized data, and the ridge regularization: $\tilde{\mathbf{S}}_{11} = \mathbf{S}_{11} + \sqrt{\log(p)/n}\mathbf{I}_p$ and $\tilde{\mathbf{S}}_{22} = \mathbf{S}_{22} + \sqrt{\log(q)/n}\mathbf{I}_q$. Also, τ_1 and τ_2 are tuning parameters which are chosen by cross-validation. See Safo et al. (2018) for more details. For subsequent canonical directions $\boldsymbol{\alpha}_i$, and $\boldsymbol{\beta}_i$, $i > 1$, the authors solved the above sparse optimization problem on deflated data.

1.4 Prediction with SELPCCA

CCA and SELPCCA are unsupervised multivariate methods, with their main goal of finding canonical vectors that maximize the overall dependency structure between two views. In some biomedical applications, it is usually the case that an outcome variable, say $\mathbf{y}$, is available and a specific interest might be to investigate how these canonical variates are related

to the outcome of interest. For this purpose, one can build regression models to associate the outcome with these canonical variates. In our package, we provide `selpPredict()` for this purpose. We provide options gaussian, binomial, poisson, and survival to model continuous, categorical, count, or time-to-event data, respectively. We also provide the function `predict()` to predict out of sample data using the learned low-dimensional representations or canonical variates.

1.5 Sparse Integrative Discriminant Analysis (SIDA) for two or more views

Instead of considering the two-step CCA approach proposed above when there is a clinical outcome in addition to the views, the authors in Safo et al. (2021) developed the method, SIDA, for jointly maximizing association between two or more views while also maximizing separation between classes in each view. Although CCA is applicable to only two views, SIDA is applicable to two or more views. SIDA combines the advantages of LDA, a supervised learning method for maximizing separation between classes in a view, and CCA, an unsupervised learning method for maximizing correlation between two data types. Suppose there are $d = 1, \ldots, D$ views. Let $\mathbf{X}^d = [\mathbf{X}_1^d, \mathbf{X}_2^d, \ldots, \mathbf{X}_K^d]$, $\mathbf{X}^d \in \Re^{n \times p_d}, \mathbf{X}_k^d \in \Re^{n_k \times p_d}, k = 1, \ldots, K,\ d = 1, 2, \ldots, D$ be a concatenation of the K classes in the d-th view. Let $\mathbf{S}_b^d$ and $\mathbf{S}_w^d$ be the between-class and within-class covariances for the d-th view. Let $\mathbf{S}_{dj}, j < d$ be the cross-covariance between the d-th and j-th views. Define $\mathcal{M}^d = \mathbf{S}_w^{d^{-1/2}} \mathbf{S}_b^d \mathbf{S}_w^{d^{-1/2}}$ and $\mathcal{N}_{dj} = \mathbf{S}_w^{d^{-1/2}} \mathbf{S}_{dj} \mathbf{S}_w^{j^{-1/2}}$. The authors solved the following optimization problem for the basis discriminant vectors $\mathbf{\Gamma}^d$:

$$\max_{\mathbf{\Gamma}^1, \cdots, \mathbf{\Gamma}^D} \rho \sum_{d=1}^{D} \text{tr}(\mathbf{\Gamma}^{d\text{T}} \mathcal{M}^d \mathbf{\Gamma}^d) + \frac{2(1-\rho)}{D(D-1)} \sum_{d=1, d \neq j}^{D} \text{tr}(\mathbf{\Gamma}^{d\text{T}} \mathcal{N}_{dj} \mathbf{\Gamma}^j \mathbf{\Gamma}^{j\text{T}} \mathcal{N}_{jd} \mathbf{\Gamma}^d)$$
$$\text{s.t } \text{tr}(\mathbf{\Gamma}^{d\text{T}} \mathbf{\Gamma}^d) = K - 1.$$

Of note, ρ controls the influence of separation or association in the optimization problem. The second term sums the unique pairwise squared correlations and weights them by $\frac{D(D-1)}{2}$ so that the sum of the squared correlations is one. The authors showed that the nonsparse basis discriminant directions for the d-th view, $\widetilde{\mathbf{\Gamma}}^d$, that maximize both associations and separations are given by the eigenvectors corresponding to the eigenvalues ($\mathbf{\Lambda}^d$) that iteratively solve the following eigensystems:

$$\begin{aligned} \left(c_1 \mathcal{M}^1 + c_1 \mathcal{M}^{1^{\text{T}}} + c_2 \overline{\mathcal{N}}_{1j} + c_2 \overline{\mathcal{N}}_{1j}^{\text{T}}\right) \mathbf{\Gamma}^1 &= \mathbf{\Lambda}_1 \mathbf{\Gamma}^1, \\ &\vdots \\ \left(c_1 \mathcal{M}^D + c_1 \mathcal{M}^{1^{\text{T}}} + c_2 \overline{\mathcal{N}}_{Dj} + c_2 \overline{\mathcal{N}}_{Dj}^{\text{T}}\right) \mathbf{\Gamma}^D &= \mathbf{\Lambda}_D \mathbf{\Gamma}^D, \end{aligned} \tag{2}$$

where $c_1 = \rho$ and $c_2 = \frac{2(1-\rho)}{D(D-1)}$, and $\overline{\mathcal{N}}_{dj} = \sum_{d,j}^{D} \mathcal{N}_{dj} \mathbf{\Gamma}^j \mathbf{\Gamma}^{j^{\text{T}}} \mathcal{N}_{jd},\ d, j = 1, \ldots D, j \neq d$ sums all unique pairwise correlations of the d-th and the j-th views.

For sparsity or smoothness, they considered the following optimization problems:

$$
\begin{aligned}
&\min_{\boldsymbol{\Gamma}^1} \mathcal{P}(\boldsymbol{\Gamma}^1) \qquad \text{s.t} \qquad \|(c_1\mathcal{M}^1 + c_1{\mathcal{M}^1}^{\mathrm{T}} + c_2\overline{\mathcal{N}}_{1j} + c_2\overline{\mathcal{N}}_{1j}^{\mathrm{T}})\widetilde{\boldsymbol{\Gamma}}^1 - \widetilde{\boldsymbol{\Lambda}}_1\boldsymbol{\Gamma}^1\|_\infty \leq \tau_1 \\
&\qquad \vdots \\
&\min_{\boldsymbol{\Gamma}^D} \mathcal{P}(\boldsymbol{\Gamma}^D) \qquad \text{s.t} \qquad \|(c_1\mathcal{M}^D + c_1{\mathcal{M}^D}^{\mathrm{T}} + c_2\overline{\mathcal{N}}_{Dj} + c_2\overline{\mathcal{N}}_{Dj}^{\mathrm{T}})\widetilde{\boldsymbol{\Gamma}}^D - \widetilde{\boldsymbol{\Lambda}}_D\boldsymbol{\Gamma}^D\|_\infty \leq \tau_D. \qquad (3)
\end{aligned}
$$

The authors considered two forms of penalty term $\mathcal{P}(\boldsymbol{\Gamma}^d)$, depending on whether sparsity only (data-driven) or sparsity and smoothness (knowledge-driven) is desired: $\mathcal{P}(\boldsymbol{\Gamma}^d) = \sum_{i=1}^{p_d} \|\boldsymbol{\gamma}_i^d\|_2$, or $\mathcal{P}(\boldsymbol{\Gamma}^d) = \eta \sum_{i=1}^{p_d} \|\boldsymbol{\gamma}_i^{\mathcal{L}_n}\|_2 + (1-\eta)\sum_{i=1}^{p_d} \|\boldsymbol{\gamma}_i\|_2$. In the latter, $\boldsymbol{\gamma}_i^{\mathcal{L}_n}$ is the i-th row of the matrix product $\mathcal{L}_n\boldsymbol{\Gamma}^d$, and $\mathcal{L}_n$, is the normalized Laplacian of a graph, which is view-dependent. Essentially, the first term in this penalty acts as a smoothing operator for the weight matrices $\boldsymbol{\Gamma}^d$ so that variables that are connected within the d-th view are encouraged to be selected or neglected together. This was termed SIDANet (SIDA for structured or network data). SIDANet is applicable when there exists prior biological information in the form of variable-variable connections, which guides the detection of connected variables that maximize both separation and association.

2 Visualizations of discriminant scores and canonical correlation variates

Once the discriminant vectors or canonical correlation vectors have been estimated using SIDA/SIDANet or SELPCCA respectively, the scores for each view, representing projections of each view onto these vectors can be estimated. We provide `DiscriminantPlots()` and `CorrelationPlots()` to visualize these scores. The `DiscriminantPlots()` function can be used to visualize the first (assuming only two classes) or the first and second discriminant scores (assuming > two classes). Each point on this plot represent a score for an individual. Discriminant plots can showcase how well the discriminant vectors separate the classes. Correlation plots on the other hand can be used to visualize the strength of association between pairs of views as well as the separation of the classes. Figure S1 demonstrates sample figures that can be generated by our `DiscriminantPlots()` and `CorrelationPlots()` in `mvlearnR`. We provide different color options for the graphs.

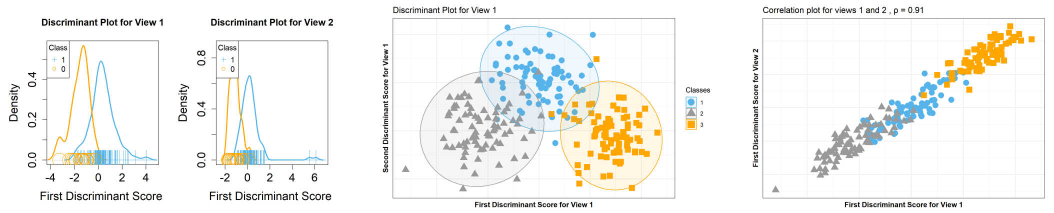

Figure S1: Sample discriminant plots for two classes (left panel), for three classes (middle panel) and correlation plot (right panel). Discriminant plots can demonstrate whether the discriminant vectors separate the classes. Correlation plots demonstrate the strength of association between pairs of views. It also shows how well the classes are separated.

3 Visualizations of variables selected

3.1 Relevance Network

Relevance Network (RN) (Butte et al., 2000, González et al., 2012) have been proposed to visualize pairwise relationships of variables in integrative analysis. Here, the nodes represent variables, and edges represent variable associations. To construct RN, the correlation matrix of pairwise associations is obtained from the data. Then, for a specific cutoff (say 0.7), if the estimated correlation coefficients is greater (in absolute value) than this cutoff, an edge is drawn between these two variables; otherwise, no edge is drawn and these two variables are deemed not associated for this threshold. Further, variables/nodes with no link are not represented in the RN. The cutoff is used to make the graph less dense, especially when the number of variables is high. RN have potential to shed lignt into the complex associations between different types of data. We provide the function `networkPlot()` to visualize the pairwise relationships of variables selected by SELPCCA, SIDA, and SIDANet. We follow ideas in González et al. (2012) to construct the RN for SELPCCA, SIDA, and SIDANet. In particular, instead of computing the Pearson correlation coefficients between each pair of variables in each view separately, we construct bipartite graph (or bigraph) where variables/nodes from view $\mathbf{X}^d$ are connected to variables/nodes from view $\mathbf{X}^j$, $d \neq j, d, j = 1, \ldots, D$. Similar to González et al. (2012), we construct the bipartite graphs using a pairwise similarity matrix directly obtained from the outputs of our integrative analysis methods.

Suppose we have applied SELPCCA to two views and we have obtained the canonical loadings $\widehat{\boldsymbol{\alpha}}_l$ and $\widehat{\boldsymbol{\beta}}_l$, $l = 1, \ldots, L$, for views 1 and 2, respectively. For $l > 2$, it is likely that within a view, different variables will have nonzero coefficients for each canonical loading. Therefore, we deem a variable to be selected if it has a nonzero coefficient in at least one canonical loading. Let p' and q' be the number of selected variables in views 1 and 2, respectively. Let $\widehat{\mathbf{A}} = (\widehat{\boldsymbol{\alpha}}_1^{\mathrm{T}}, \ldots, \widehat{\boldsymbol{\alpha}}_L^{\mathrm{T}})^{\mathrm{T}} \in \Re^{p' \times L}$ be a concatenation of all L canonical loadings for view 1. Let $\widehat{\mathbf{B}} = (\widehat{\boldsymbol{\beta}}_1^{\mathrm{T}}, \ldots, \widehat{\boldsymbol{\beta}}_L^{\mathrm{T}})^{\mathrm{T}} \in \Re^{q' \times L}$ be defined similarly for view 2. Given data with these selected variables, we construct the canonical variates: $\mathbf{U} = \mathbf{X}^1_{selected}\widehat{\mathbf{A}}$ and $\mathbf{V} = \mathbf{X}^2_{selected}\widehat{\mathbf{B}}$. To construct the $p' \times q'$ similarity matrix for views 1 and 2, we first project data for the selected variables to $\mathbf{U}$, for view 1, $\mathbf{V}$ for view 2, or to a combination of $\mathbf{U}$ and $\mathbf{V}$. Since $\mathbf{X}^1$ and $\mathbf{X}^2$ are analyzed simultaneously in CCA, the projection of the data onto a combination of $\mathbf{U}$ and $\mathbf{V}$ seems natural. Thus, we define $\mathbf{Z} = \mathbf{U} + \mathbf{V}$. As noted in González et al. (2012), the $\mathbf{Z}$ variables are closest to $\mathbf{X}^d$ and $\mathbf{X}^j$. Then, for the ith variable $X_i^1 \in \mathbf{X}^1_{selected}$ (similarly $X_i^2 \in \mathbf{X}^2_{selected}$) and the lth component $\mathbf{z}_l \in \mathbf{Z}$, we compute the scalar inner product $x_{il}^d = \langle X_i^d, \mathbf{z}_l \rangle, d = 1, 2; j = 1 \ldots p' (\text{or } \; q'),\; l = 1, \ldots, L$. Assuming that the variables X_i^d, and $\mathbf{z}_l$ are standardized to have variance 1, $x_{il}^d = \langle X_i^d, \mathbf{z}_l \rangle = \mathrm{cor}(X_i^d, \mathbf{z}_l)$. We compute the similarity matrix $\mathbf{M}$ between views 1 and 2 as $\mathbf{M} = \mathbf{x}^1 \mathbf{x}^{2'\mathrm{T}} \in \Re^{p' \times q'}$, where $\mathbf{x}^1$ is a $p' \times L$ matrix with the ilth entry x_{il}^1 and $\mathbf{x}^2$ is a $q' \times L$ matrix with the ilth entry x_{il}^2. Each entry in the similarity matrix is between -1 and 1.

We construct the similarity matrix for SIDA and SIDANet in a similar fashion. Of note, in SIDA and SIDANet, $l = K - 1$. Although, the $l_{2,1}$ norm encourages the same variables to be selected across all $K - 1$ components, empirically, this is not usually the case. Therefore, we obtain overall variable selection as described above. We obtain the discriminant vectors for the dth and jth views as $\mathbf{U} = \mathbf{X}^1_{selected}\widehat{\boldsymbol{\Gamma}}^d$ and $\mathbf{V} = \mathbf{X}^2_{selected}\widehat{\boldsymbol{\Gamma}}^j$, respectively. Given $\mathbf{U}$ and $\mathbf{V}$, we compute the similarity matrix for SIDA and SIDANet in a similar fashion.

We generate the relevance network from the similarity matrix. The nodes of the graph

represent variables for the pairs of views, and edges represent the correlations between pairs of variables. The function `networkPlot()` in `mvlearnR` can be used to generate relevance networks. Please refer to the bottom left and right panels of Figure S2 for sample relevance network plots. Dashed and solid lines indicate negative and positive correlations respectively. Circle nodes are View 1 variables, and rectangular nodes are View 2 variables. We provide an option to tune the network through a correlation cutoff. For instance in the middle right panel of Figure S2, we only show graph for variables with correlations greater than 0.9. The plot suggest that variable V1031 from View 2 is highly correlated with variable V31 from View 1. Since there is no edge between variable V38 and variables V1037, it indicates that the correlation between these pairs of variables is smaller than 0.9.

3.2 Variable importance plots

We provide the function `VarImportancePlot()` [Top left panel of Figure S2] to visualize the weights (in absolute value) of the loadings. Since the loadings are standardized to have unit norm, a variable with larger weight contributes more to the association between the views (for SELPCCA) or to the association between the views and discrimination of classes within each view (SIDA and SIDANet). We only show the top 20 variables and their weights but one can view data matrix for all variables.

3.3 Loadings plots

We provide the function `LoadingsPlot()` [Top right panel of Figure S2] to plot discriminant and canonical correlation vectors. These graphs are useful for visualizing how selected variables from SIDA/SIDANet and SELPCCA contribute to the first and second discriminant (for SIDA and SIDANet) or canonical correlation (for SELPCCA) vectors. Variables farther from the origin and close to the first or second axis have higher impact on the first or second discriminant/canonical vectors, respectively. Variables farther from the origin and between both first and second axes have similar higher contributions to the first and second discriminant/canonical correlation vectors. In both situations, for SIDA and SIDANet, this suggests that these variables contribute more to the separation of classes and association of views. For SELPCCA, this suggests that these variables contribute more to the association between the two views. This plot can only be generated for classification and association problems with 3 or more classes (SIDA and SIDANet), or for CCA problems with two or more canonical correlation vectors requested (i.e. ncancorr > 1 for SELPCCA). The angle between two vectors also give an indication of how the two variables are correlated. In particular, vectors that are close to each other suggests that the variables have high positive correlation. Vectors that are about 90 degrees indicate that the two variables are uncorrelated. Vectors that have an angle close to 180 degrees indicate that the two variables have negative correlation.

4 Visualization of loadings and scores simultaneously

Biplots are useful for representing both loadings and discriminant scores/canonical correlation variates. We present biplots for each view and between views. In particular, we provide the function `WithinViewBiplot()` to visualize the scores and loadings for each view separately [Figure 1, Bottom left panel]. We also provide the function `BetweenViewBiplot()` to graph scores and loadings for pairs of views. The scores are the sum of scores for the two

views (Refer to Section on relevance network for more explanation). Please refer to Figure 1, Bottom right panel for a sample biplot between views. In this graph, dashed red vectors represent loadings plot for the second view. And solid black vectors represent loadings plot for the first view. Please refer to section 3.3 for a brief discussion on interpreting loadings plots.

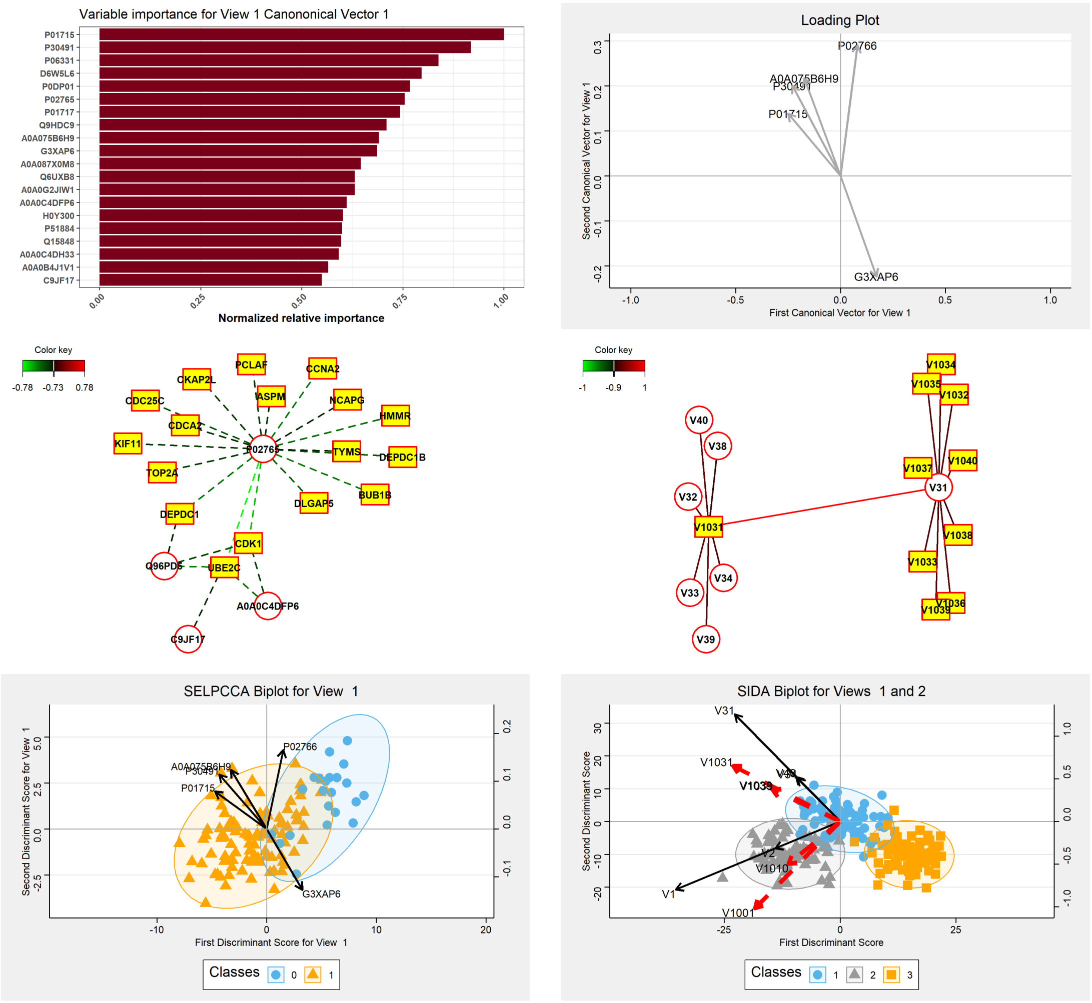

Figure S2: Sample plots for visualizing variables within and between views. *Top left panel*: variable importance plot showing the relative importance of each variable. Variables with largest absolute loadings rank high. The weights for each variable is normalized relative to the largest weight. *Top right panel*: Loading plots. We provide loading plots for each view to demonstrate the relationships between pairs of variables within each view. Variables that are farther from the origin have higher impact on the axis the vector is close to. Vectors that are close to each other suggest that the pairs of variables are correlated positively. Vectors with an angle of about 180 degrees suggest that the variables have negative correlation. Vectors with a 90 degree angle between them suggest that the two variables are not correlated. *Middle left and right panels*: Relevance network showing variable-variable connections between pairs of views with correlations > 0.73 [left panel] and > 0.9 [right panel]. Dashed and solid lines indicate negative and positive correlations, respectively. Color key gives the direction of the associations where green indicates negative correlations and red shows positive correlations. *Bottom left panel*: Within-view biplot. This plot shows the scores and loadings together for a specific view. Bottom right panel: Between-view biplot. This plot shows the scores and loadings from pairs of views together. The scores are the sum of scores for each view. Solid and dashed lines represent vectors for Views 1 and 2, respectively. In this example, variable V31 (from View 1) has a higher correlation with variables V1031 and V1038 from View 2 compared to Variable V1001 from View 2. This finding is supported by the edge between V31 and V1038 and V1031 in the network plot but no edge between V31 and V1001 given a correlation cutoff of 0.9.

5 Demonstration of SELPCCA and SELPCCA Predict on COVID-19 Data

```
#load data
#load data
data("COVIDData")

#make omics data numeric
Proteomics= apply(as.matrix(COVIDData[[1]]), 2, as.numeric)
RNASeq= apply(as.matrix(COVIDData[[2]]), 2, as.numeric)
Clinical= COVIDData[[3]]

table(Clinical$DiseaseStatus)
```

Figure S3: Import COVID-19 data

```
set.seed(1234)
stratified <- Clinical %>%
  group_by(DiseaseStatus) %>%
  sample_frac(size = .9)

#train data
Proteomics2=cbind.data.frame(Clinical[,1], Proteomics)
Proteomics.Train=Proteomics2[Proteomics2[,1] %in% stratified$ID, ]
Clinical.Train=Clinical[Clinical[,1] %in% stratified$ID, ]

RNASeq2=cbind.data.frame(Clinical[,1], RNASeq)
RNASeq.Train=RNASeq2[RNASeq2[,1] %in% stratified$ID, ]

#test data
set.diff=setdiff(Clinical[,1],stratified$ID)
Proteomics.Test=Proteomics2[Proteomics2[,1] %in% set.diff, ]
RNASeq.Test=RNASeq2[RNASeq2[,1] %in% set.diff, ]
Clinical.Test=Clinical[Clinical[,1] %in% set.diff, ]

#order data to ensure that the rows are the same
Proteomics.Train=Proteomics.Train[order(Proteomics.Train[,1]),]
Proteomics.Test=Proteomics.Test[order(Proteomics.Test[,1]),]

RNASeq.Train=RNASeq.Train[order(RNASeq.Train[,1]),]
RNASeq.Test=RNASeq.Test[order(RNASeq.Test[,1]),]

Clinical.Train=Clinical.Train[order(Clinical.Train[,1]),]
Clinical.Test=Clinical.Test[order(Clinical.Test[,1]),]
```

Figure S4: Train and Test splits. We split the data into 90% train and 10% test, keeping the proportion of COVID-19 and non-COVID-19 cases as in the original data.

```
#Supervised filtering- Logistic regression with B-H adjusted pvalues
X=list(Proteomics.Train[,-1], RNASeq.Train[,-1] )
Xtest.in=list(Proteomics.Test[,-1], RNASeq.Test[,-1] ) #testing data will be subsetted to keep only variables that are significant in training set
Y=Clinical.Train$DiseaseStatus.Indicator

filterOmics=filter.supervised( X,
          Y,
          method = "logistic",
          padjust=TRUE,
          adjmethod="BH",
          thresh = 0.05,
          center = FALSE,
          scale = FALSE,
          log2TransForm = FALSE,
          standardize=TRUE,
          Xtest = Xtest.in
                       )
```

	Coef <dbl>	Pval <dbl>	Keep <lgl>	View <int>
P04196	-2.302249	0.0005777604	TRUE	1
P51884	-1.833268	0.0005777604	TRUE	1
P30491;P30685;P30490;P18464;P10319;P30498;P30483;P18463;P18465;P30487;P30488;P30485	1.155279	0.0012273746	TRUE	1
A0A0C4DFP6;Q9NQ79-2;Q9NQ79;Q9NQ79-3;Q5T4F6	-2.242493	0.0012464527	TRUE	1
C9JB55	-2.553349	0.0012464527	TRUE	1
P02765;C9JV77	-1.492872	0.0012464527	TRUE	1
V9GYM3;P02652;V9GYE3;V9GYG9	-1.549336	0.0012464527	TRUE	1
Q6UXB8;Q6UXB8-2	-1.660778	0.0013409020	TRUE	1
Q96PD5;Q96PD5-2	-1.498597	0.0014091137	TRUE	1
D6W5L6;P07988;H0Y7V6	1.359330	0.0017111773	TRUE	1

1-10 of 10 rows

	Coef <dbl>	Pval <dbl>	Keep <lgl>	View <int>
ASPM	2.334767	0.0003191221	TRUE	2
BIRC5	2.130810	0.0003191221	TRUE	2
BUB1	2.533314	0.0003191221	TRUE	2
BUB1B	2.368631	0.0003191221	TRUE	2
CCNB2	2.456306	0.0003191221	TRUE	2
CDC20	1.941254	0.0003191221	TRUE	2
CDC25C	2.448140	0.0003191221	TRUE	2
CDCA2	2.465166	0.0003191221	TRUE	2
CDCA5	2.400132	0.0003191221	TRUE	2
CDCA8	2.423595	0.0003191221	TRUE	2

10 rows

Figure S5: Supervised filtering with logistic regression. Example output table from supervised filtering showing Top 10 (sorted by p-values) molecules with estimated coefficients (Coef), P-value (Pval), whether the variable is kept or filtered out (Keep), and the view (View). View 1 is for proteomics data, and View 2 is for RNASeq data.

```
volcanoPlot(filterOmics)
```

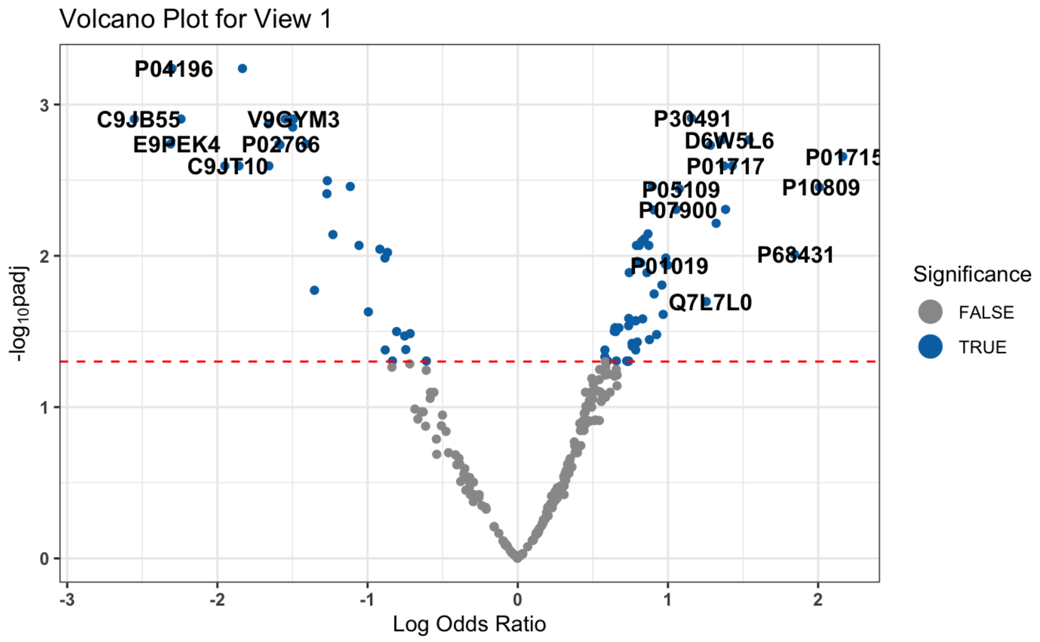

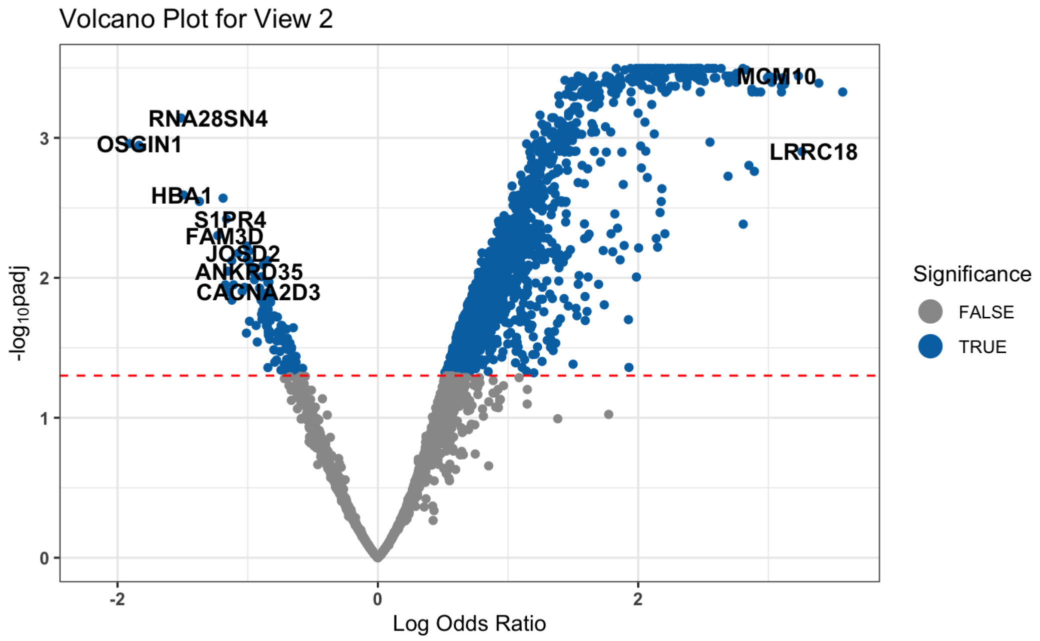

Figure S6: Volcano plots for View 1 (Proteins) and View 2 (Genes).

```
umapPlot(filterOmics)
```

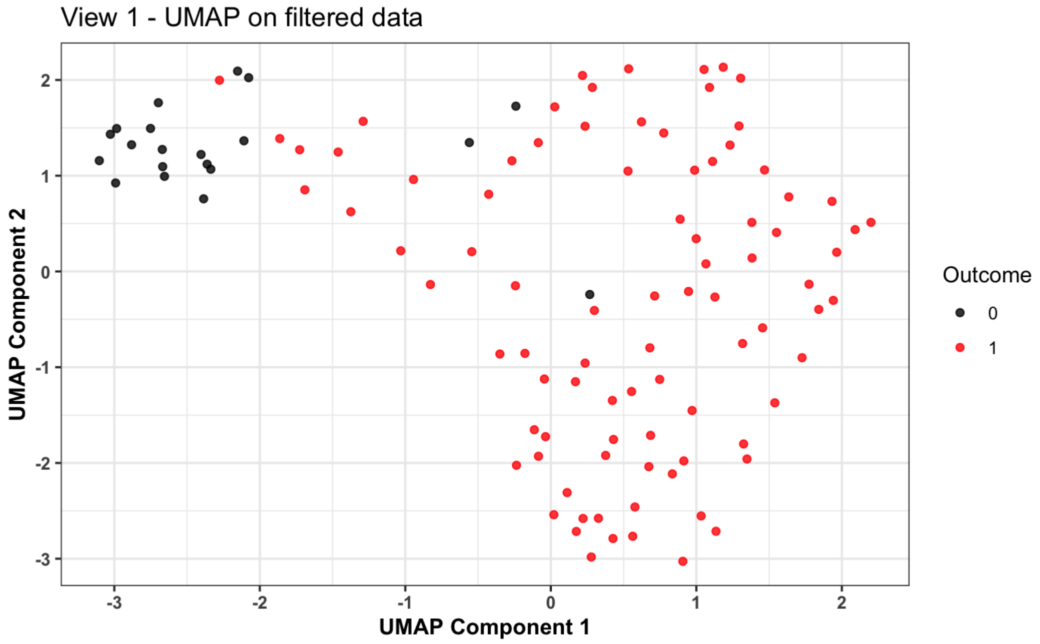

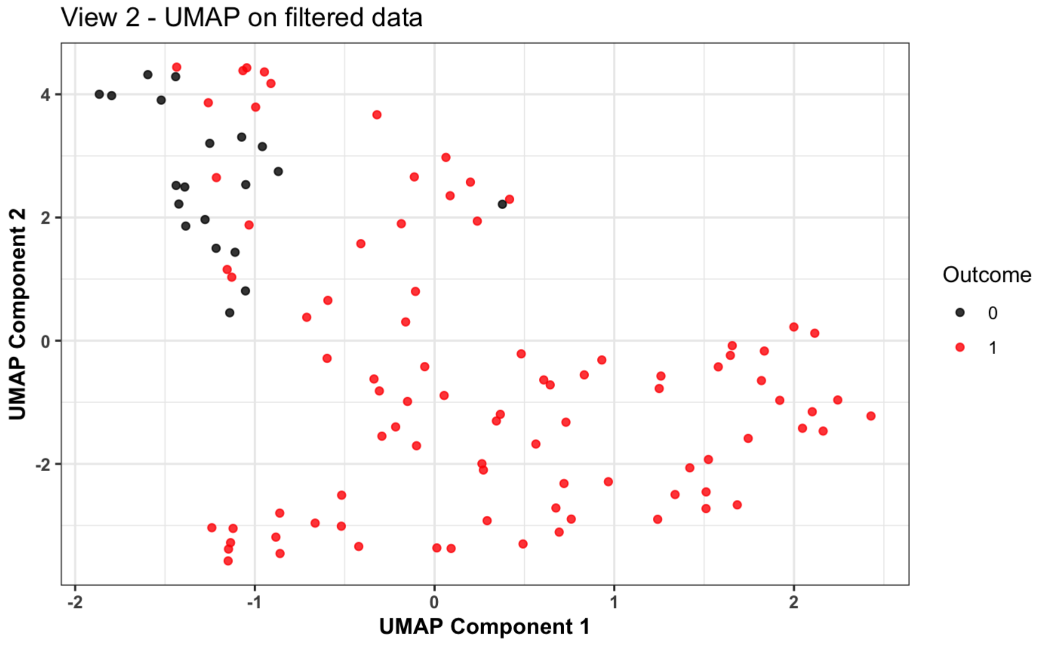

Figure S7: UMAP plots for View 1 (Proteins) and View 2 (Genes) on filtered data.

```r
#can use simulated data
#data("selpData")
#Xdata1 <- selpData[[1]]
#Xdata2 <- selpData[[2]]

#We use the filtered data from above  and obtain first and second canonical correlation vectors
#We use cross-validation to choose tuning parameters for sparsity
Xdata1 <- filterOmics$X[[1]] #proteomics
Xdata2 <- filterOmics$X[[2]] #RNASeq

mycvselpcca=cvselpscca(Xdata1, Xdata2, ncancorr=2)
```

```
## [1] "Current iteration is 1"
## [1] "Current iteration is 2"
## [1] "Applying optimal tuning parameter on whole data"
## [1] "Current Iteration Is: 1"
## [1] "Current Iteration Is: 2"
## [1] "Number of non-zero Xdata1 or View 1: 78 54"
## [1] "Number of non-zero Xdata2 or View 2: 32 9"
## [1] "Corr(Xdata1*alpha,Xdata2*beta): 0.636 0.599"
## [1] "Sparse CCA CovStructure used is: Iden"
```

Figure S8: Fitting SELPCCA with cross-validation on training data to choose optimal hyperparameters and to obtain overall canonical correlation vectors on whole data based on the optimal hyperparameter. We use the function `cvselpscca()`.

```
VarImportancePlot(mycvselpcca)
```

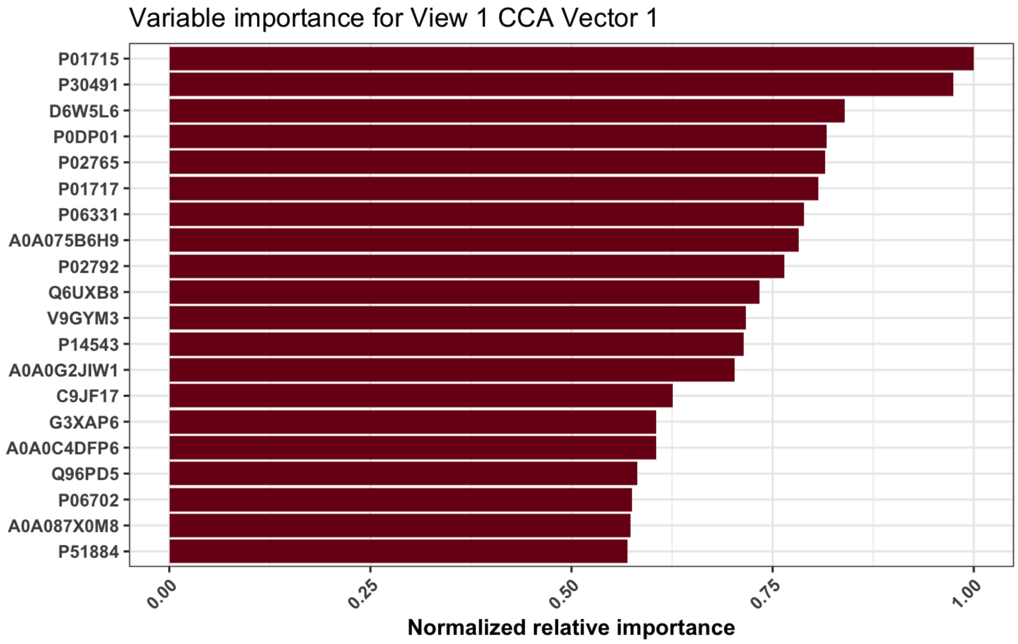

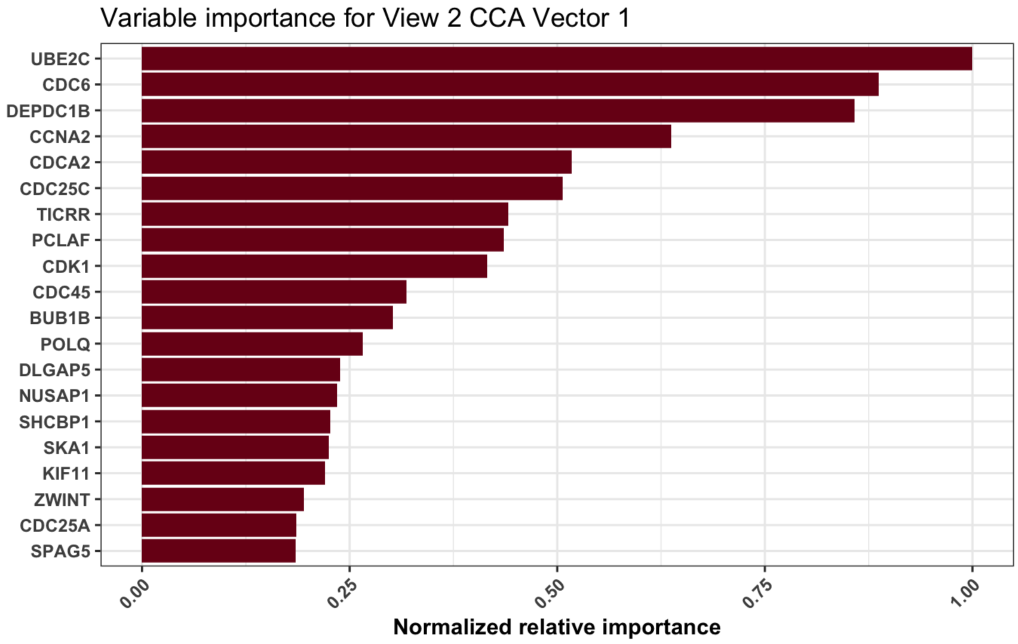

Figure S9: Variable important plots for the first canonical vector for View 1 (Proteins) and View 2 (Genes). Top 20 variables with largest absolute weights for first canonical correlation vector from application of SELPCCA are shown. For proteins, Uniprot IDs are shown on variable importance plot.

```r
#train data
Xdata1 <- filterOmics$X[[1]]
Xdata2 <- filterOmics$X[[2]]
Y=filterOmics$Y
Xdata=list(Xdata1, Xdata2)

hatalpha=list(mycvselpcca$hatalpha[,1],mycvselpcca$hatbeta[,1])

DiscriminantPlots(Xdata, Y,hatalpha,method.used="SELPCCA")
```

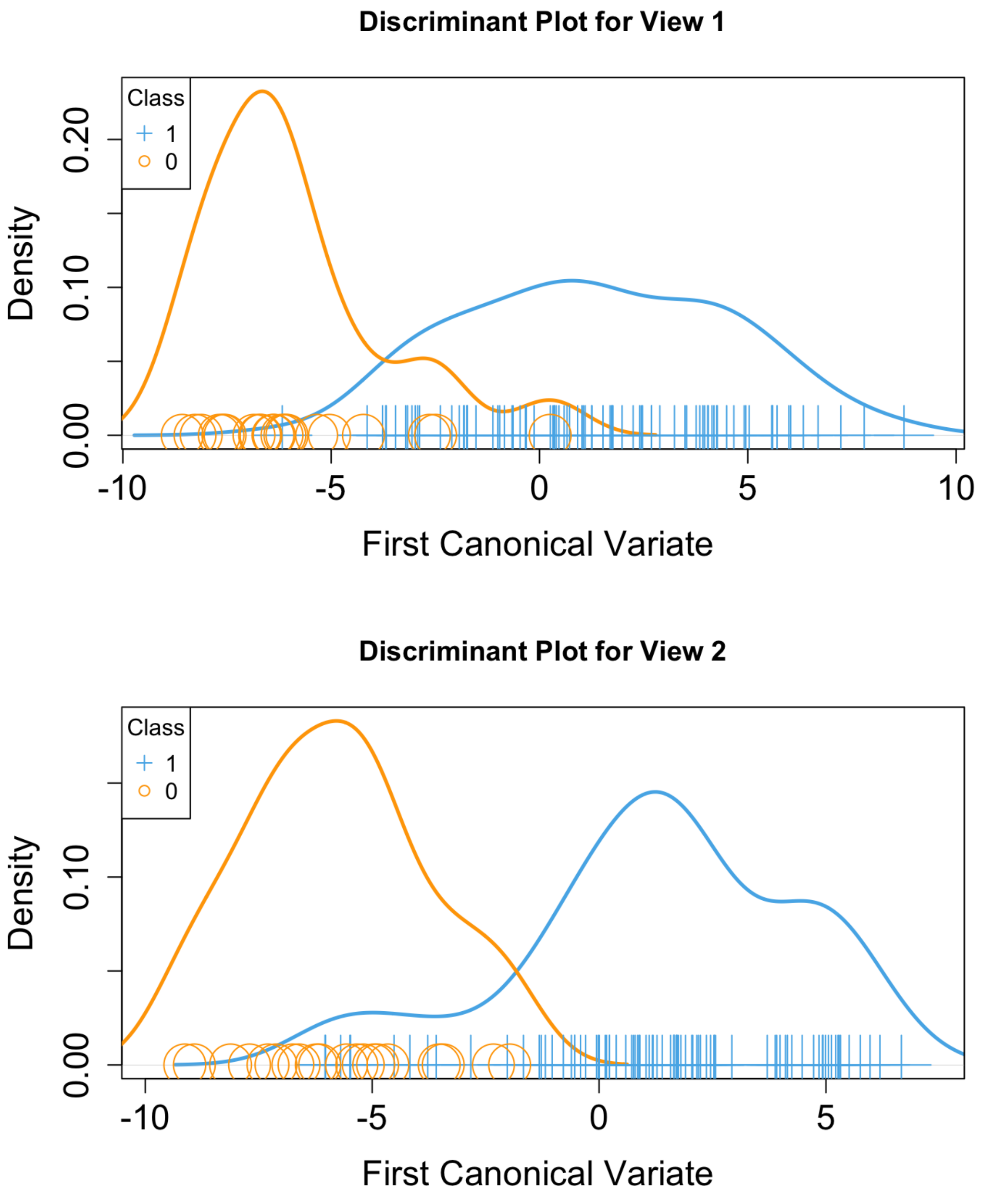

Figure S10: Discriminant plots for SELPCCA based on training data. Top Panel: proteins and Bottom panel: genes. We use the function `DiscriminantPlots()` to generate these plots. Class 0 is non-COVID-19 samples. Class 1 is COVID-19 samples.

```r
#test data
Xtest1 <- filterOmics$Xtest[[1]] #proteomics
Xtest2 <- filterOmics$Xtest[[2]] #RNASeq
Xtest.in=list(Xtest1, Xtest2)
Ytest.in=Clinical.Test$DiseaseStatus.Indicator

DiscriminantPlots(Xtest.in, Ytest.in, hatalpha,method.used="SELPCCA")
```

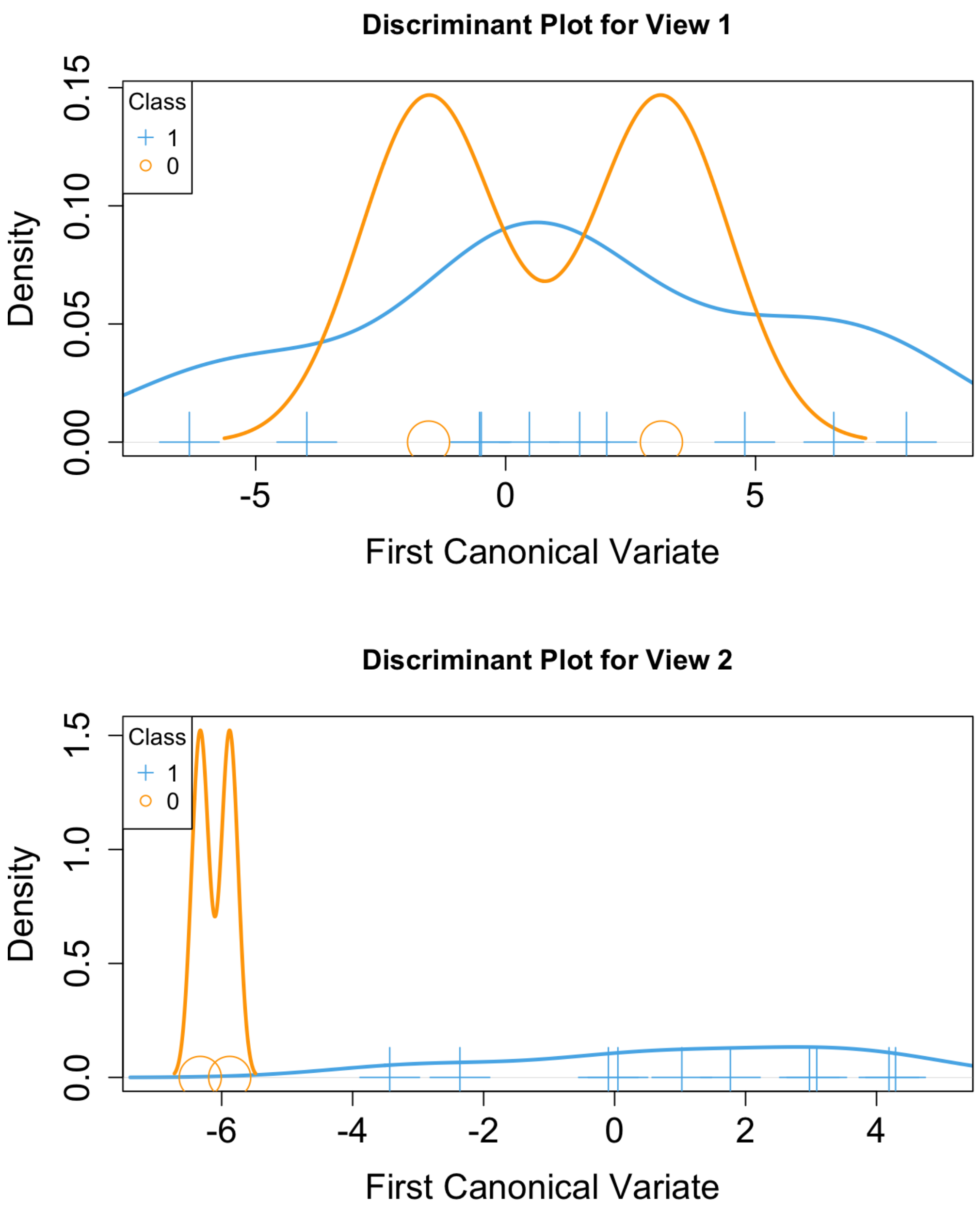

Figure S11: Discriminant plots for SELPCCA based on testing data. Top Panel: proteins and Bottom panel: genes. We use the function `DiscriminantPlots()` to generate these plots. Class 0 is non-COVID-19 samples. Class 1 is COVID-19 samples.

```
Y=Clinical.Train$DiseaseStatus.Indicator
WithinViewBiplot(mycvselpcca,Y,Xtest=NULL,keep.loadings = c(7,3))
```

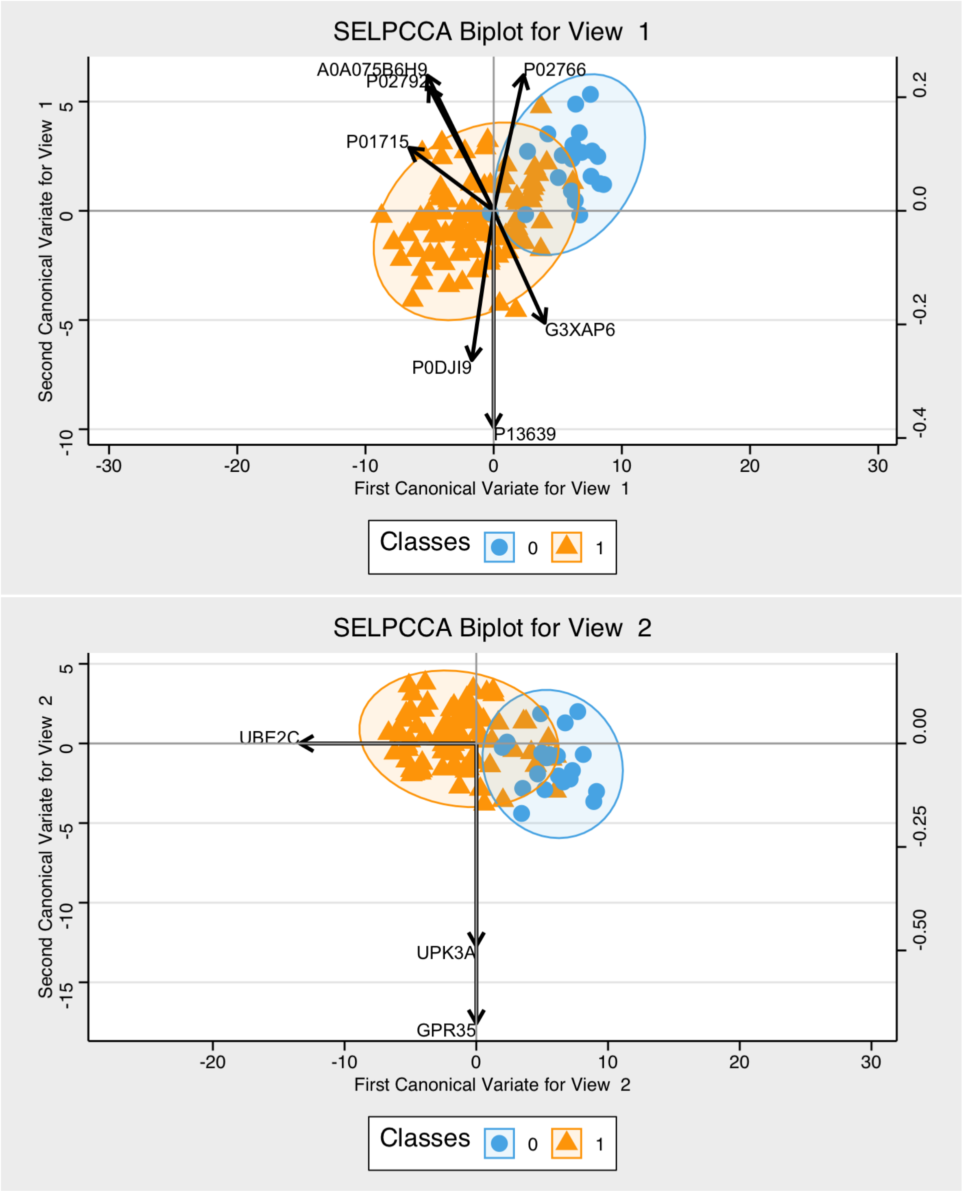

Figure S12: Within-view biplots. Biplots are useful for representing both loadings plot and discriminant scores/canonical correlation variates. We provide the function `WithinViewBiplot()` to visualize the scores and loadings for each view separately. In this plot, we only show labels for top 7 proteins and 3 genes with largest absolute weights. Top Panel: Protein IDs shown are loaded on both the first and second canonical vectors. Protein ID A0A075B6H9 appears to be highly correlated with P02792. Bottom Panel: the gene UBE2C is loaded on the first canonical vector; genes UPK3A and GPR35 are loaded on the second canonical vector. Class 0 is non-COVID-19 samples. Class 1 is COVID-19 samples.

```
Y=Clinical.Train$DiseaseStatus.Indicator


BetweenViewBiplot(mycvselpcca,Y,Xtest=NULL, keep.loadings = c(7,3))
```

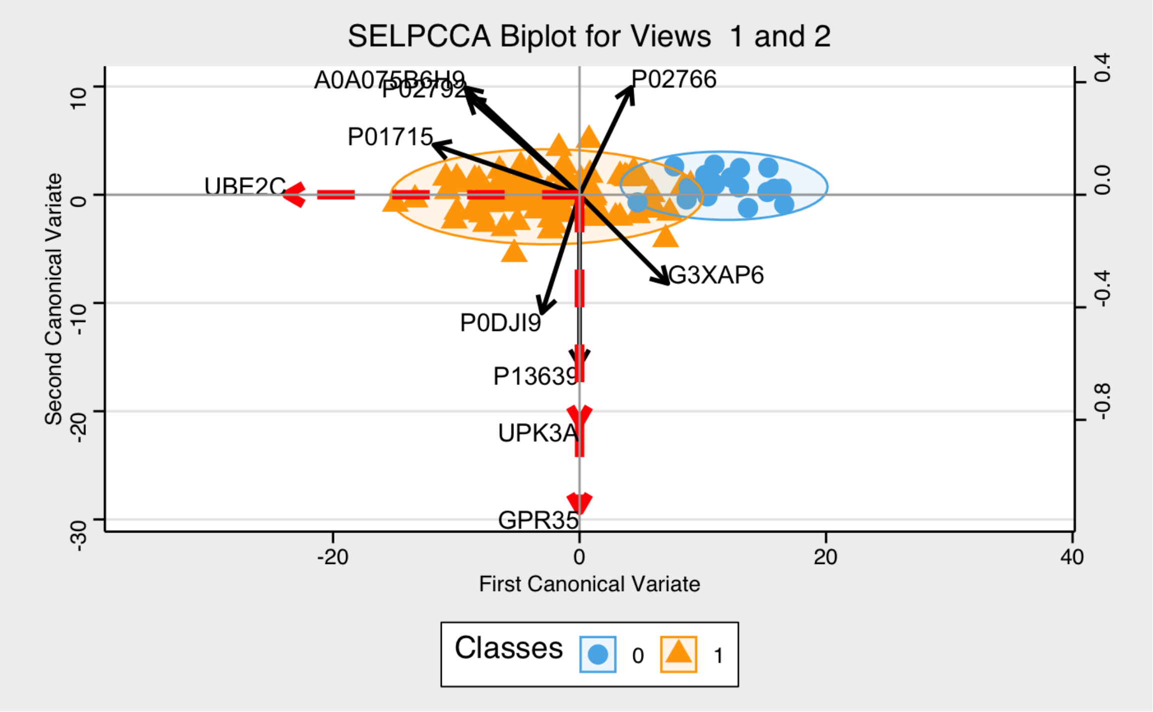

Figure S13: Between-view biplots are useful for visualizing biplots for both views. This plot allows us to assess how genes and proteins are related. Solid black vectors represent loading plots for the first view (proteins). Dashed red vectors represent loadings plot for the second view (genes). We generate this plot with the function `BetweenViewBiplot()`. In this plot, we only show labels for the top 7 proteins and 3 genes with largest absolute weights. The protein Immunoglobulin lambda variable 3-1 (UID P01715) appears to be positively correlated with the gene UBE2C. The protein P02792 appears to be negatively correlated with the genes UPK3A and GPR35. Class 0 is non-COVID-19 samples. Class 1 is COVID-19 samples.

```
networkPlot(mycvselpcca, cutoff=0.58)
```

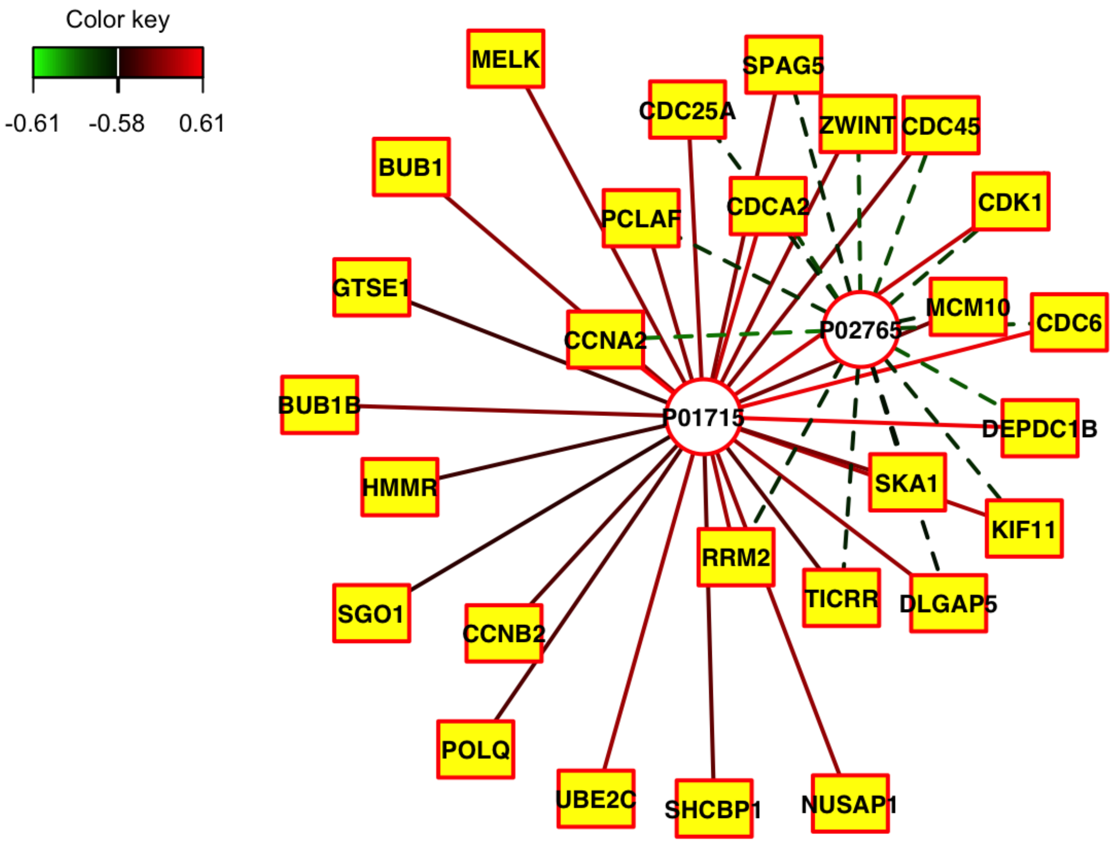

Figure S14: Relevance network plot. The nodes of the graph represent variables for the pairs of views, and edges represent the correlations between pairs of variables. Dashed and solid lines indicate negative and positive correlations, respectively. Circle nodes are View 1 variables (proteins), and rectangular nodes are View 2 variables (genes). We show edges with correlations at least 0.58. The plot suggest that the protein P01715 is highly positively correlated with many genes, and the protein P02765 is highly negatively correlated with many genes.

```r
#Use results from cvselpcca. One can also use selpPredict() to train the model by setting fitselpCCA=NULL.

Y.train=Clinical.Train$DiseaseStatus.Indicator
myresult=selpscca.pred(Xdata1, Xdata2, Y.train,fitselpCCA=mycvselpcca, family="binomial",showProgress=T)
```

```
## Fitting SELPCCA Model
## Fitting Prediction Model
```

```r
print(summary(myresult[["mod.fit"]]))
```

```
##
## Call:
## stats::glm(formula = factor(Y) ~ ., family = stats::binomial,
##     data = selp.dat)
##
## Coefficients:
##             Estimate Std. Error z value Pr(>|z|)
## (Intercept)   7.1934     2.4013   2.996  0.00274 **
## X1_1          0.7856     0.3128   2.511  0.01203 *
## X1_2         -0.6519     0.4968  -1.312  0.18946
## X2_1          0.9732     0.3945   2.467  0.01362 *
## X2_2          0.2319     0.4886   0.475  0.63502
## ---
## Signif. codes:  0 '***' 0.001 '**' 0.01 '*' 0.05 '.' 0.1 ' ' 1
##
## (Dispersion parameter for binomial family taken to be 1)
##
##     Null deviance: 103.500  on 107  degrees of freedom
## Residual deviance:  16.346  on 103  degrees of freedom
## AIC: 26.346
##
## Number of Fisher Scoring iterations: 9
```

Figure S15: Prediction with the first two canonical variates from SELPCCA. Results suggest that the first canonical variate for view 1 and view 2 are significantly associated with COVID-19 status (p-value < 0.05). That is, the first canonical variates for views 1 (proteins) and 2 (genes) are able to discriminate between those with and without COVID-19.

```r
##Train Performance Metrics
newpredictions=predict(myresult, newdata=Xdata1, newdata2=Xdata2)
Y.pred=round(newpredictions$pred.mod)
train.metrics=PerformanceMetrics(Y.pred,Y.train,family='binomial',isPlot=TRUE)
```

	Metrics <dbl>
Accuracy	0.96296296
Error.rate	0.03703704
Sensitivity	0.97727273
Specificity	0.90000000
Matthews.Correlation.Coefficient	0.87727273
Balanced.Accuracy	0.93863636
Balanced.Error.Rate	0.06136364
F1.Score	0.97727273
False.Discovery.Rate	0.02272727
Positive.Predictive.Value	0.97727273

10 rows

Figure S16: Train Performance metrics. We use the function `PerformanceMetrics()` to obtain these metrics. Top panel: Train predictions.

```
##Test Performance Metrics
Xtest1 <- filterOmics$Xtest[[1]] #proteomics
Xtest2 <- filterOmics$Xtest[[2]] #RNASeq
Y.test=Clinical.Test$DiseaseStatus.Indicator
newpredictions=predict(myresult, newdata=Xtest1, newdata2=Xtest2)
Y.pred=round(newpredictions$pred.mod)
test.metrics=PerformanceMetrics(Y.pred,Y.test,family='binomial',isPlot=TRUE)
```

	Metrics <dbl>
Accuracy	0.8333333
Error.rate	0.1666667
Sensitivity	1.0000000
Specificity	0.0000000
Matthews.Correlation.Coefficient	0.0000000
Balanced.Accuracy	0.5000000
Balanced.Error.Rate	0.5000000
F1.Score	0.9090909
False.Discovery.Rate	0.1666667
Positive.Predictive.Value	0.8333333

1–10 of 10 rows

Figure S17: Test Performance metrics. We use the function `PerformanceMetrics()` to obtain these metrics. Top panel: Train predictions.

6 Demonstration of SIDA on COVID-19 Data

```
#can use simulated data
# data("sidaData")
# Xdata <- sidaData[[1]]
# Y <- sidaData[[2]]
# Xtestdata <- sidaData[[3]]
# Ytest <- sidaData[[4]]

#We use the filtered data from above and obtain discriminant vectors that maximize association
#between gene and protein data while discriminating between those with and without COVID-19.
#We use cross-validation to choose variables
Xdata1 <- filterOmics$X[[1]] #proteins
Xdata2 <- filterOmics$X[[2]] #RNASeq
Y=filterOmics$Y+1  # class membership needs to be numeric, coded as 1, 2, ....
Xdata=list(Xdata1, Xdata2)

Xtest1 <- filterOmics$Xtest[[1]] #proteomics
Xtest2 <- filterOmics$Xtest[[2]] #RNASeq
Xtest.in=list(Xtest1, Xtest2)
Ytest.in=Clinical.Test$DiseaseStatus.Indicator


#We apply the function cvSIDA() to obtain estimated SIDA discriminant vectors, correlation coefficients, and vari
ables potentially contributing to the association of the views and the discrimination between samples within each
view.

fit.cvsida <- cvSIDA(Xdata, Y,
                     Xtestdata = Xtest.in,
                     Ytest = Ytest.in+1, plotIt = F)
```

```
## [1] "Getting tuning grid values"
## [1] "Completed at time"
## Time difference of 8.269799 secs
## Begin 5 -folds cross-validation
## [1] "Cross-validation completed at time"
## Time difference of 1.211282 mins
## [1] "Getting Results......"
## Estimated Test Classification Error is 0.08333333
## Estimated Train Classification Error is 0.01851852
## Estimated Test Correlation is 0.06680775
## Estimated Train Correlation is 0.4129295
## Number of nonzero coefficients in view 1 is 26
## Number of nonzero coefficients in view 2 is 23
## [1] "Total time used is"
## Time difference of 1.555339 mins
```

```
#From implementing SIDA, we observed that 26 proteins and 23 genes have nonzero coefficients, which suggests that
these proteins and genes maximize both correlation between the proteomics and RNASeq data (estimated correlation
is 0.42) as well as separation between those with and without COVID-19.
```

Figure S18: Fitting SIDA with cross-validation on training data to choose optimal hyperparameters and to obtain overall discriminant vectors based on the optimal hyperparameter. We use the function `cvSIDA()`.

```
VarImportancePlot(fit.cvsida)
```

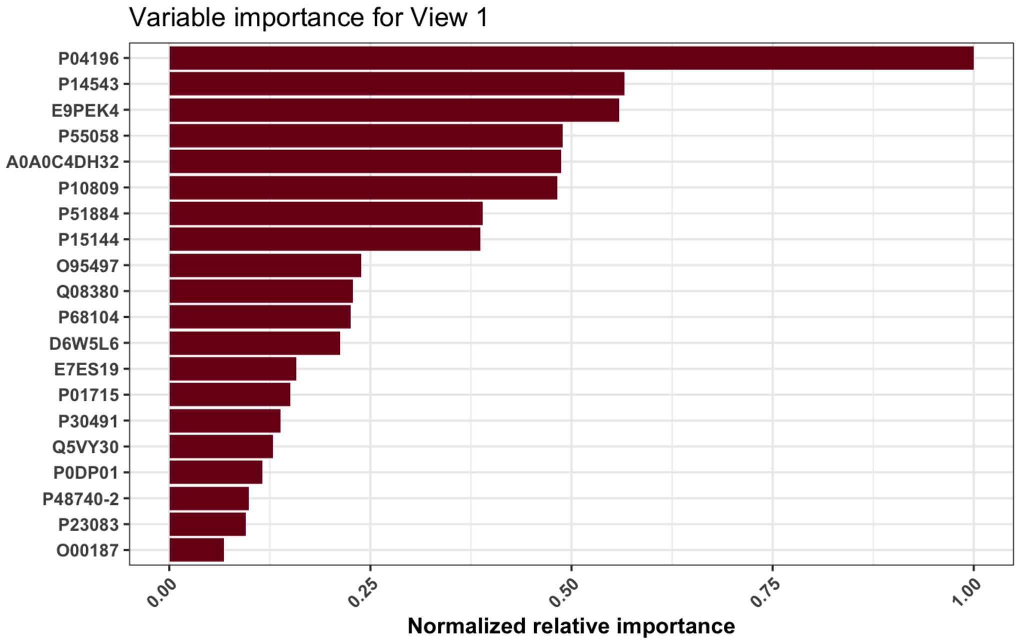

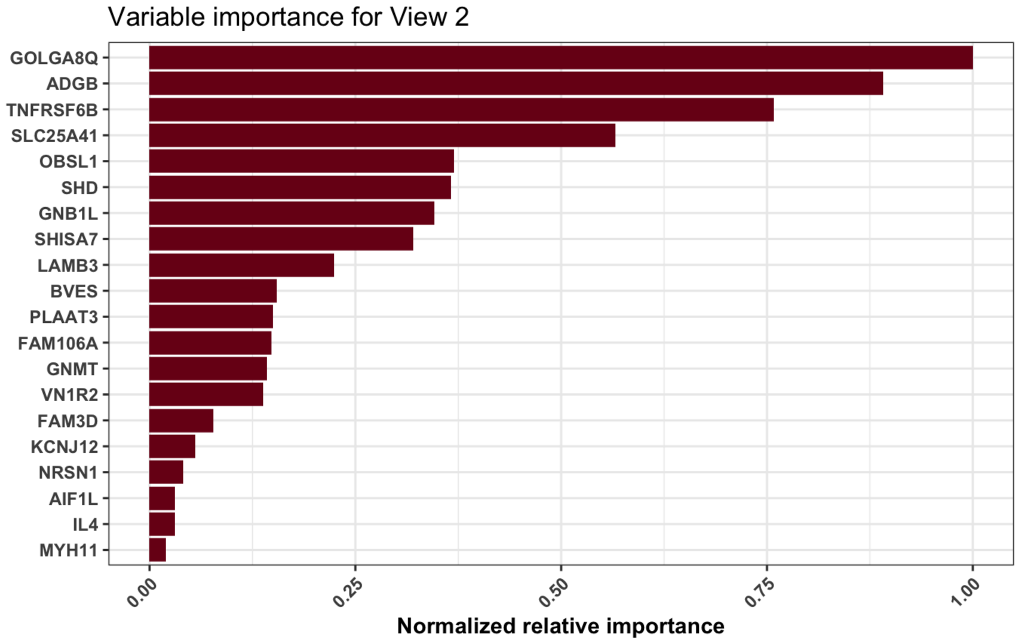

Figure S19: Variable important plots for View 1 (Proteins) and View 2 (Genes) after implementing SIDA. Top 20 variables with largest absolute weights are shown. For proteins, Uniprot IDs are shown on variable importance plot.

```
#train data
Xdata1 <- filterOmics$X[[1]]
Xdata2 <- filterOmics$X[[2]]
Y=filterOmics$Y  # class membership needs to be numeric, coded as 1, 2, ....
Xdata=list(Xdata1, Xdata2)

DiscriminantPlots(Xdata, Y, fit.cvsida$hatalpha)
```

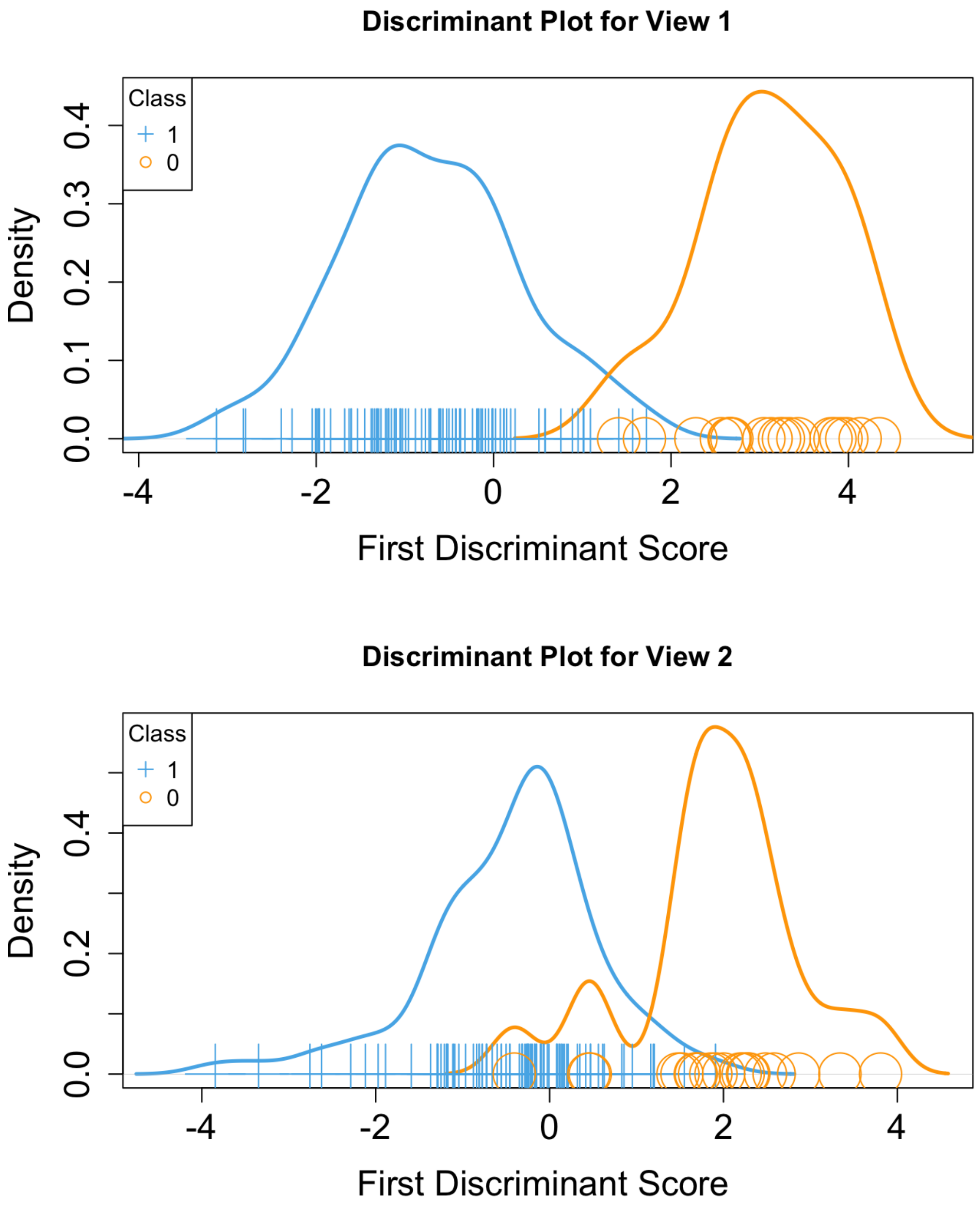

Figure S20: Discriminant plots based on training data. Top Panel: proteins and Bottom panel: genes. We use the function `DiscriminantPlots()` to generate these plots. Class 0 is non-COVID-19 samples. Class 1 is COVID-19 samples.

```r
#test data
Xtest1 <- filterOmics$Xtest[[1]] #proteomics
Xtest2 <- filterOmics$Xtest[[2]] #RNASeq
Xtest.in=list(Xtest1, Xtest2)

DiscriminantPlots(Xtest.in, Ytest.in, fit.cvsida$hatalpha)
```

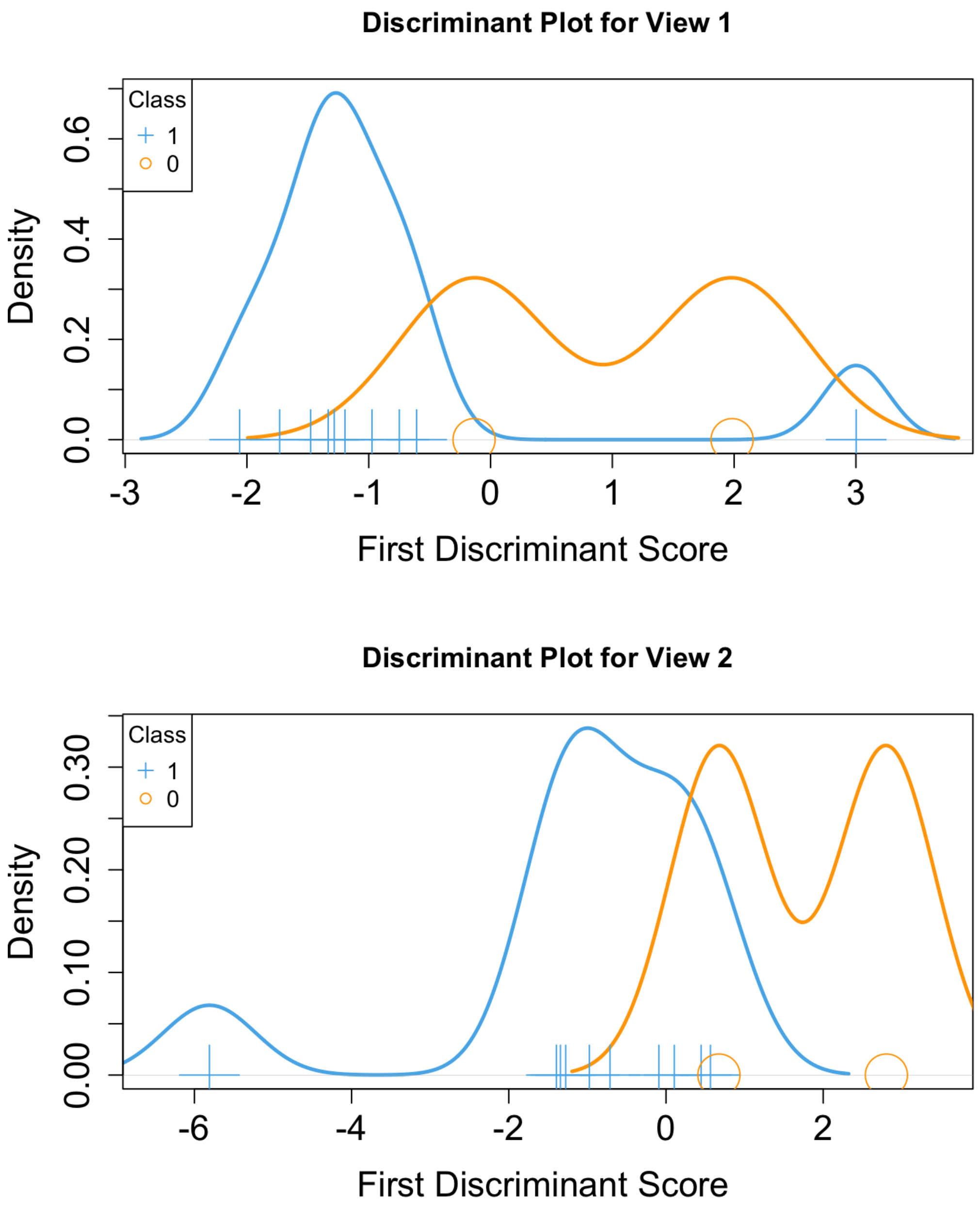

Figure S21: Discriminant plots based on testing data. Top Panel: proteins and Bottom panel: genes. We use the function `DiscriminantPlots()` to generate these plots. Class 0 is non-COVID-19 samples. Class 1 is COVID-19 samples.

```
Xdata1 <- filterOmics$X[[1]]
Xdata2 <- filterOmics$X[[2]]
Y=filterOmics$Y
Xdata=list(Xdata1, Xdata2)

CorrelationPlots(Xdata, Ytest=Y, fit.cvsida$hatalpha)
```

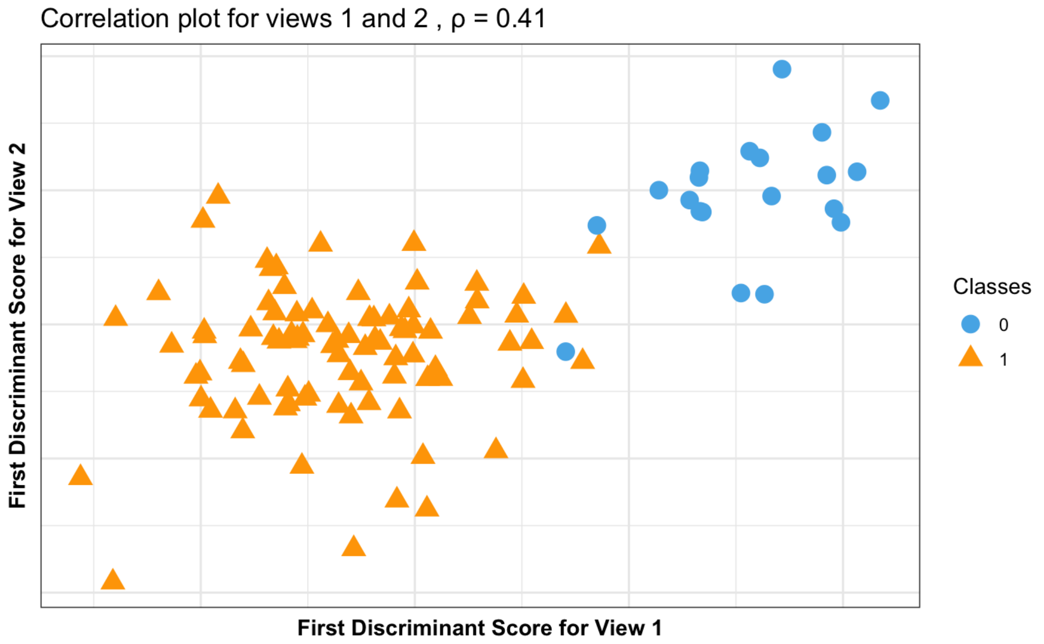

Figure S22: Correlation plots based on training data. We use the function `CorrelationPlots()` to generate these plots. Class 0 is non-COVID-19 samples. Class 1 is COVID-19 samples.

```
networkPlot(fit.cvsida,cutoff=.1)


#The plot suggest that the gene FAM3D is negatively  correlated with many proteins (e.g. PO2766, P30491, Q08380),
and positively correlated with proteins such as A0ADC4DFP6, E9PEK4, P04196, D6W5L6.
```

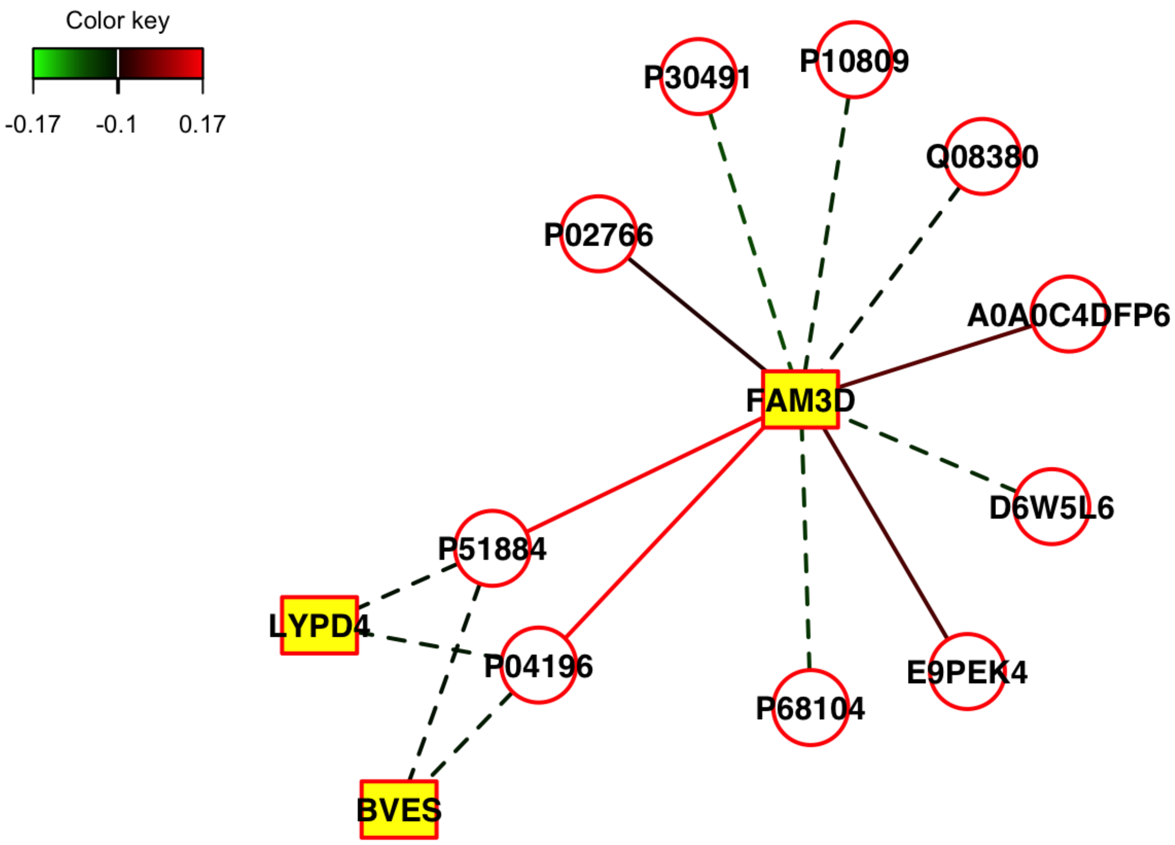

Figure S23: Relevance network plot for SIDA. The nodes of the graph represent variables for the pairs of views, and edges represent the correlations between pairs of variables. Dashed and solid lines indicate negative and positive correlations, respectively. Circle nodes are View 1 variables (proteins), and rectangular nodes are View 2 variables (genes). We show edges with correlations at least 0.1. The plot suggest that the gene FAM3D is negatively correlated with many proteins (e.g. PO2766, P30491, Q08380), and positively correlated with proteins such as A0ADC4DFP6, E9PEK4, P04196, D6W5L6.

```r
#train metrics
Y.pred=fit.cvsida$PredictedClass.train-1 #to get this in 0 and 1
Y.train=filterOmics$Y
train.metrics=PerformanceMetrics(Y.pred,Y.train,family='binomial',isPlot=FALSE)
print(train.metrics)
```

	Metrics<br><dbl>
Accuracy	0.98148148
Error.rate	0.01851852
Sensitivity	0.98863636
Specificity	0.95000000
Matthews.Correlation.Coefficient	0.93863636
Balanced.Accuracy	0.96931818
Balanced.Error.Rate	0.03068182
F1.Score	0.98863636
False.Discovery.Rate	0.01136364
Positive.Predictive.Value	0.98863636

10 rows

Figure S24: Performance metrics. We use the function `PerformanceMetrics()` to obtain these metrics.

```r
#obtain predicted class
Y.pred=fit.cvsida$PredictedClass-1
test.metrics=PerformanceMetrics(Y.pred,Ytest.in,family='binomial',isPlot=FALSE)
print(test.metrics)
```

	Metrics <dbl>
Accuracy	0.91666667
Error.rate	0.08333333
Sensitivity	0.90000000
Specificity	1.00000000
Matthews.Correlation.Coefficient	0.77459667
Balanced.Accuracy	0.95000000
Balanced.Error.Rate	0.05000000
F1.Score	0.94736842
False.Discovery.Rate	0.00000000
Positive.Predictive.Value	1.00000000

10 rows

Figure S25: Performance metrics. We use the function `PerformanceMetrics()` to obtain these metrics.

Purpose	Functions	Description
Importing Data	`data(COVIDData)`	Multiomics data pertaining to COVID-19
	`data(selpData)`	Simulated data example for SELPSCCA
	`data(sidaData)`	Simulated data example for SIDA
	`data(sidanetData)`	Simulated data example for SIDANet
Filtering	`filter.unsupervised()`	Unsupervised Filtering. Options are variance and IQR filtering. Default is variance.
	`filter.supervised()`	Supervised Filtering. Filtering options are linear, logistic, t-test, and Kruskal-Wallis.
Unsupervised data integration	`cvselpscca()`	Performs n-fold cross validation to select optimal tuning parameters for SELPCCA based on training data.
	`cvtunerange()`	Obtain upper and lower bounds of tuning parameters of SELPCCA for each canonical correlation vector.
	`multiplescca()`	Estimates the sparse canonical correlation vectors for fixed tuning parameters.
Supervised data integration	`selpscca.pred()`	A two-step approach for integration and prediction based on SELPCCA. Performs n-fold cross validation to select optimal tuning parameters for SELPCCA based on training data. Then uses the results to build a GLM or survival model for a pre-specified outcome.
	`predict.SELPCCA()`	Prediction for out-of-sample data for SELPCCA predict.
	`sida()`	Joint integration and discriminant analysis for fixed tuning parameters.
	`cvSIDA()`	Performs nfolds cross validation to select optimal tuning parameters for sida based on training data, which are then used with the training or testing data to predict class membership.
	`sidanet()`	Joint integration and discriminant analysis with incorporation of prior biological information (variable-variable relationships), for fixed tuning parameters.
	`cvSIDANet()`	Performs nfolds cross validation to select optimal tuning parameters for sida with network information, based on training data, which are then used with the training or testing data to predict class membership.
	`sidatunerange()`	Function to provide tuning parameter grid values for each view, not including covariates, if available, for SIDA.
	`sidatunerange()`	Function to provide tuning parameter grid values for each view, not including covariates, if available, for SIDANet.
	`sidaclassify()`	Classification approach for SIDA and SIDANet
Visualizations	`volcanoPlot()`	Wrapper function for volcano plots of the results after supervised filtering.
	`umapPlot()`	Wrapper function to plot a UMAP of the results after supervised filtering.
	`VarImportancePlot()`	A graph of the absolute loadings for variables selected.
	`DiscriminantPlots()`	Plots discriminant scores (for SIDA) and canonical variates (for SELPCCA) for visualizing class separation.
	`CorrelationPlots()`	Plots for visualizing correlation between estimated discriminant vectors for pairwise data.
	`LoadingsPlots()`	Plots discriminant and canonical vectors to visualize how selected variables contribute to the first and second discriminant (for SIDA and SIDANet) or canonical correlation (for SELPCCA) vectors.
	`networkPlot()`	Network visualization of selected variables from integrative analysis methods.
	`BetweenViewBiplot()`	Biplots to visualize discriminant scores/ canonical variates between pairs of views.
	`WithinViewBiplot()`	Biplots for Discriminant Scores or Canonical Correlation Variates for each View.
Performance Estimation	`PerformanceMetrics()`	Estimates performance metrics for a predicted model. Currently works for binary and continuous outcomes.

Table S1: Description of current functions in `mvlearnR`

References

Butte, A. J., Tamayo, P., Slonim, D., Golub, T. R., and Kohane, I. S. (2000). Discovering functional relationships between rna expression and chemotherapeutic susceptibility using relevance networks. Proceedings of the National Academy of Sciences, 97(22):12182–12186.

González, I., Cao, K.-A. L., Davis, M. J., and Déjean, S. (2012). Visualising associations between paired 'omics' data sets. BioData mining, 5:1–23.

Hotelling, H. (1936). Relations between two sets of variables. Biometrika, pages 312–377.

Jain, S. and Safo, S. E. (2023). A deep learning pipeline for cross-sectional and longitudinal multiview data integration. arXiv preprint arXiv:2312.01238.

Safo, S. E., Ahn, J., Jeon, Y., and Jung, S. (2018). Sparse generalized eigenvalue problem with application to canonical correlation analysis for integrative analysis of methylation and gene expression data. Biometrics, 74(4):1362–1371.

Safo, S. E. and Lu, H. (2023). Scalable randomized kernel methods for multiview data integration and prediction. arXiv preprint arXiv:2304.04692.

Safo, S. E., Min, E. J., and Haine, L. (2021). Sparse linear discriminant analysis for multiview structured data. Biometrics, n/a(n/a).

Wang, H., Lu, H., Sun, J., and Safo, S. E. (2023). Interpretable deep learning methods for multiview learning. arXiv preprint arXiv:2302.07930.

Wang, J. and Safo, S. E. (2021). Deep ida: A deep learning method for integrative discriminant analysis of multi-view data with feature ranking–an application to covid-19 severity. ArXiv.

Zhang, W., Wendt, C., Bowler, R., Hersh, C. P., and Safo, S. E. (2022). Robust integrative biclustering for multi-view data. Statistical methods in medical research, 31(11):2201–2216.
